# Classification of patients with early-stage multiple sclerosis and healthy controls using kinematic analysis during a dual-task

**DOI:** 10.3389/frai.2025.1660801

**Published:** 2025-10-21

**Authors:** José Eduardo Rosseto Garotti, Danielli Souza Speciali, Raymundo Machado de Azevedo Neto, Patricia Maria de Carvalho Aguiar, Rodrigo Barbosa Thomaz, Tiago Abrão Setrak Sowmy, Guilherme Carlos Brech, Paulo Rodrigo Bazán, Elisa Harumi Kozasa

**Affiliations:** ^1^Hospital Israelita Albert Einstein, São Paulo, Brazil,; ^2^Graduate Program in Aging Sciences at Universidade São Judas Tadeu, São Paulo, Brazil

**Keywords:** multiple sclerosis, gait analysis, machine learning, cognition, dual task, working memory

## Abstract

Multiple sclerosis (MS) is the disabling neurological disease that currently most affects young people. Changes in gait significantly impact the functionality and independence of these individuals. This study aimed to differentiate between patients in the early stages of MS and healthy controls using machine learning in angular gait variables. This cross-sectional observational study included 38 participants, 19 with MS and 19 in the healthy control group (without neurological or orthopedic diseases). For movement analysis, a three-dimensional gait examination was conducted on patients with EDSS (Expanded Disability Status Scale) scores below 3.5 and healthy volunteers during normal gait and while performing a dual task (walking and performing a working memory task). An elastic net regression model was utilized to classify patients and healthy controls based on the kinematic variables. Our model achieved an AUC (area under the curve) of the ROC plot = 0.77 ± 0.21 using the average, an AUC of 0.94 ± 0.09 using the average and standard deviation, and AUC = 0.95 ± 0.09 when incorporating only the standard deviation of kinematic variables. The study suggests that utilizing angular gait analysis with machine learning methods is an effective approach to categorizing individuals with early-stage multiple sclerosis and healthy controls.

## Introduction

Multiple sclerosis (MS) is a long-lasting, immune-mediated and progressive disease, which affects the central nervous system in different ways (Moreira et al., 2022; Kuhlmann et al., 2002). Global estimates indicate around 2.8 million cases worldwide (Lakin et al., 2021; Harris, 2013) and generally present as periods of crisis followed by remissions (Rommer et al., 2020; Goodin et al., 2016). The majority of affected individuals are generally between 20 and 40 years of age (Moreira et al., 2022). This diagnosis during a professional peak results in increased unemployment and often requires specialized care, leading to significant social expenses (Ransohoff et al., 2015; Rotstein et al., 2006).

The main way to assess the severity and progression of multiple sclerosis is the Expanded Disability Status Scale (EDSS). This scale varies from 0 to 10 with increments of 0.5 points. A score of 0 means a normal neurological exam, while a score of 10 means death due to complications related to MS (Kurtzke, 1983).

Although the EDSS is clinically important, it has some limitations, as this scale is based on subjective assessments that lack sensitivity to small changes. This highlights the need to use more advanced methods to accurately evaluate gait characteristics in individuals with MS (van and Uitdehaag, 2017; Hobart et al., 2000). More sensitive methods could be used for early detection of gait deviations, leading to more efficient preventive measures, through physical therapy and other relevant treatments (van and Uitdehaag, 2017; Hobart et al., 2000). Furthermore, recent advancements in machine learning have significantly expanded the capabilities of gait analysis for pattern recognition and classification across various clinical and sport contexts, demonstrating its potential for identifying subtle functional differences (Ramírez and Gutiérrez, 2021; Özgür et al., 2024; Friedman et al., 2010; Xu et al., 2024; Xu et al., 2022). These developments often leverage statistical features, such as means and standard deviations of kinematic variables, for robust data representation and pattern discrimination (Pan et al., 2017).

Human gait is composed of two main phases: support and swing. The support stage covers about 60% of the walking cycle, starting with the initial heel strike and ending with toe off. The remaining, 40% of the cycle, begins with toe off and ends with foot strike (Socie et al., 2013).

Studies during walking tasks that cover short and long distances have shown that walking control involves multiple neural processes and coordination (Socie et al., 2013). The challenge becomes more pronounced in the early stages of the disease, as highlighted in a study by Morel et al. (2017). Individuals with MS, when compared to healthy individuals, present a reduction in speed, stride length, cadence and joint disability, indicating that angular details could also offer deeper insights into gait abnormalities in MS (Martin et al., 2006; Cameron and Lord, 2010).

A frequent result is cognitive impairment, observed in 43 to 70% of people with MS (Rao et al., 1991; Benedict et al., 2006). Although there is no direct link between cognitive differences and underlying problems, key areas such as processing speed and memory can draw on mental resources similar to those of gait (Chiaravalloti and DeLuca, 2008; Woollacott and Shumway-Cook, 2002; Demnitz et al., 2016).

Evaluating how these individuals walk while completing a second task adds more relevance to research in the real world (Lakin et al., 2021; Chiaravalloti and DeLuca, 2008; Woollacott and Shumway-Cook, 2002; Demnitz et al., 2016; Wajda et al., 2013). Research on walking associated with a cognitive task in people with minor functional limitations has not shown a significant decline in walking during dual-task situations, however, results may differ based on the complexity of the additional task (Woollacott and Shumway-Cook, 2002; Leone et al., 2015; Huxham et al., 2001; Hamilton et al., 2009; Liparoti et al., 2019).

The angular impacts of major joints during walking have not been extensively studied, especially in double-phase scenarios (Kurtzke, 1983; Woollacott and Shumway-Cook, 2002; Demnitz et al., 2016; Wajda et al., 2013; Leone et al., 2015; Huxham et al., 2001; Hamilton et al., 2009; Liparoti et al., 2019). However, we did not find a comprehensive joint assessment of these factors used for categorization in the existing literature (Martin et al., 2006; Woollacott and Shumway-Cook, 2002; Corradini et al., 1997).

While kinematic gait analysis and dual-task paradigms (Socie et al., 2013) have been increasingly utilized to investigate motor impairments in MS, a significant gap remains in studies specifically targeting early-stage MS patients and their subtle gait deviations (Morel et al., 2017; Martin et al., 2006). Moreover, although mean kinematic values are commonly analyzed (Martin et al., 2006; Cameron and Lord, 2010), the potential of variability metrics, such as standard deviation (SD), and their combination with mean values in classifying early MS based on gait patterns using machine learning has been largely overlooked. This study addresses these specific gaps by incorporating both mean and standard deviation of angular gait variables, offering a possible more nuanced and accurate approach to differentiate early-stage MS patients from healthy controls, which is often challenging in routine clinical assessment.

The aim of this study was to verify whether it is possible to classify patients in the early stages of multiple sclerosis and healthy controls into different groups, using angular gait data, both during normal gait and during dual-task; we also checked the most relevant variables for this classification and their relationship with the expanded scale of disability status.

## Methods

The study was performed at the Movement Study Laboratory of Hospital Israelita Albert Einstein (HIAE), with approval granted by the Ethics Committee of the HIAE (number 57428416.2.0000.0071).

### Participants

The sample of this study consisted of 38 participants, with 19 individuals diagnosed with multiple sclerosis (MS group) and 19 healthy controls (control group). The average age± s.d. in the MS group was 35.95 years (± 5.79), while in the control group it was 33.22 years (± 6.76), with no statistically significant difference between the groups (*p* = 0.264; Mann–Whitney test). Regarding body mass index (BMI), the MS group had an average of 25.22 kg/m^2^ (± 3.99), and the control group averaged 24.42 kg/m^2^ (± 8.40), also showing no significant difference (*p* = 0.889; Mann–Whitney test). The average years of education were 17.5 (± 3.26) in the MS group and 20.0 (± 3.76) in the control group, with no statistical difference between the groups (*p* = 0.083; Mann–Whitney test). Both groups showed a predominance of females (84% in the MS group and 79% in the control group), with a similar distribution between the groups (*p* = 1.000; Fisher’s exact test). The demographic characteristics demonstrate a relatively homogeneous sample, enhancing the comparability of the groups in subsequent analyses.

This cross-sectional observational study included 38 individuals, with 19 diagnosed with MS and EDSS scores between 0 and 3.5, and 19 forming the healthy control group (without neurological or orthopedic diagnosis). During the entire period, treatments had to be continued and no evidence of active disease was found. All participants were previously diagnosed MS according to McDonald’s diagnostic criteria (Polman et al., 2011) and recruited from the MS Center of the Integrated Neurology Program at HIAE.

As inclusion criteria we considered being aged 18 to 60 years old. In the specific case of the MS group, the disease was stabled for at least 6 months. The exclusion criteria were having cognitive limitations that make it difficult to understand the informed consent form.

Gait data from 75 volunteers were collected over a 2-year interval, 38 of which were matched into the control groups and the MS group according to age (with a variation of plus or minus 3 years), gender and education. This rigorous matching was crucial to ensure the highest possible comparability between the groups, thus enhancing the reliability of our analysis despite the limited sample size inherent to this specific patient cohort.

### Experimental task

**Figure 1:**
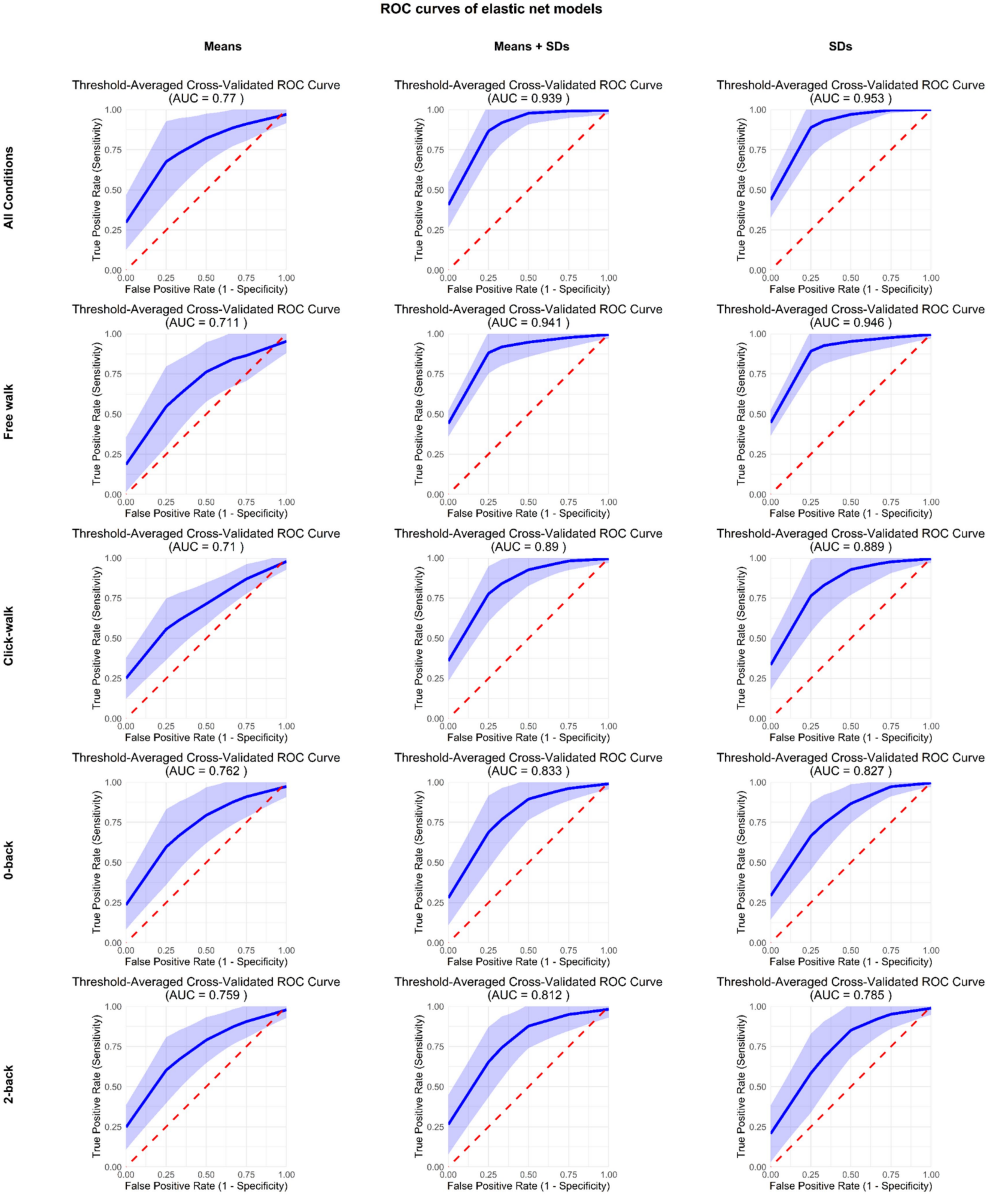
Average ROC curves generated through threshold averaging across 25 cross-validation folds (5-fold × 5 repeats) for each of the models. The blue line represents the mean true positive rate at each false positive rate threshold, while the light blue band indicates the variability (±1 SD) in model performance across different data splits. The dashed red line represents random classification performance. On the left side the models using only the mean metrics; in the middle, the models using both mean and SD variables; on the right the ROC curve for models which only used the SD variables.

Gait data were collected using the Vicon^®^ system composed of 10 infrared cameras, generating three-dimensional data on the movement of the lower limbs from reflectors attached to the individuals’ skin. Figure 1 provides an illustrative overview of the data collection setup, detailing the polystyrene spherical reflective markers (A), the infrared camera system (B), and the schematic representation of anatomical regions demarcated by reflective markers adhered to the volunteers’ skin, selected based on the plug-in gait protocol (C). One hundred and fifty angular kinematic data were collected. All gait acquisitions were performed at the Einstein Movement Study Laboratory at HIAE. This high-precision optoelectronic system was specifically chosen for its capacity to accurately capture subtle kinematic variations, which are critical for identifying early gait impairments often not detectable by conventional clinical assessments in individuals with early-stage MS (Morel et al., 2017; Martin et al., 2006). For comprehensive details regarding specific equipment models, calibration procedures, and the exact data collection protocols, please refer to the Supplementary material.

Cognitive performance was assessed using the n-back task (Hastie et al., 2009), which evaluates working memory (Friedman et al., 2010; Rooney et al., 2020; Shanahan et al., 2018) by asking participants to remember the number presented in audio form 2 positions ago. This task was chosen due to the high prevalence of working memory deficits in individuals with Multiple Sclerosis, even in early stages of the disease, which significantly impact their daily functioning (Rao et al., 1991; Benedict et al., 2006; Chiaravalloti and DeLuca, 2008). The inclusion of two n-back conditions (0-back and 2-back) allowed for a graded assessment of cognitive load during gait. The 0-back task served as a low-demand condition, primarily assessing sustained attention and basic auditory processing. Conversely, the 2-back task presented a higher cognitive load, requiring continuous updating, monitoring, and inhibition of irrelevant information within working memory. Additionally, the ‘Click-Walk’ task was incorporated to serve as a motor-attentional control, isolating the effect of auditory stimulus processing during walking with minimal working memory engagement. This comprehensive selection of tasks enables a nuanced investigation into the interplay between cognitive demands and motor control, which is critical for identifying subtle gait impairments in MS patients under real-world conditions (Woollacott and Shumway-Cook, 2002; Wajda et al., 2013; Rooney et al., 2020). The experimental design of the paradigm was in blocks adopting the following instructions:

1. Free walk: carried out to collect baseline gait parameters. The participant was instructed to walk at a comfortable speed for at least 1 min.
2. Dual task: While walking at a comfortable speed, the participant had to perform n-back tasks. The dual task condition consisted of three distinct blocks lasting 20 s and interspersed with pauses (15 s) to collect the baseline:

- 0-back block: upon listening to a sequence from 0 to 9, the participant should have pressed a response button whenever they heard the number 0.
- 2-back block: upon listening to a sequence from 1 to 9, the participant should press a response button whenever the current number was the same as the one mentioned 2 previous positions.
- Click-Walk block: the participant had to press a response button whenever they listened to the ‘click’ command.

Participants walked at a comfortable speed. The average time for data collection at the Einstein Movement Study Laboratory was 1 h and 40 min.

### Statistical methods

Descriptive statistics were used to identify the absolute and relative angular frequencies obtained, in addition to reporting average, medians and standard deviations (Rooney et al., 2020). The variables involved, in each body segment, were average (AVG) and range of motion (Range). These data were applied to a classification model, seeking to evaluate the ability to classify between controls and patients. Elastic Net regression models were used (Friedman et al., 2010; Shanahan et al., 2018). Elastic Net (Ramírez and Gutiérrez, 2021) is a regularization technique widely used in linear regression models within the field of Machine Learning, especially in contexts with many variables and high multicollinearity between them (Rooney et al., 2020). This method was specifically chosen for its ability to perform automatic feature selection and handle correlated variables. It also provides a more interpretable outcome, which was crucial for identifying the most discriminating gait parameters from our high-dimensional kinematic dataset, aligning with our study’s primary objective. The model parameters were optimized using Machine Learning methodology (Ramírez and Gutiérrez, 2021; Hamilton et al., 2009). As implemented in the glmnet package, version 4.1–8 (Friedman et al., 2010), two main parameters are optimized: alpha, a value from 0 to 1 which defines the balance between Ridge (when alpha is 0) and Lasso (when alpha is 1) regression; and lambda, a positive number which is the weight of the penalty correction. The models were trained using a 5-fold cross validation with 5 repetitions and parameter tuning was performed with alpha ranging from 0 to 1 (in 0.01 intervals) and lambda ranging from 0 to 50 (in 0.05 intervals). A fixed seed was defined for reproducibility (see the analysis code in the Supplementary material). The performance metric is the cross-validated area under the curve (AUC) of the ROC plot. Because the sample size is small, we did not create a separate test set, only validation sets for the cross-validation procedure. The gait variables were standardized by subtracting the mean and dividing by the standard deviation to allow using the relation between the weights of the different gait variables in the model as indicator of the relative importance of the variables for the classification. The classification models were generated using 5 subsets of the variables: one with all the data in all phases of the experiment, and another with data from each experimental condition separately (Click-Walk, 0-back, 2-back and the Free walk condition). Additionally, models were estimated using only average gait metric values, only standard deviation gait metric values, and including both average and standard deviation of the metrics.

## Results

### Classification using the average

Using the average the best model achieved AUC = 0.77 ± 0.21 (average ± SD). These results indicate that when using 600 gait kinematic variables (150 variables, 75 on the right side of the body and 75 on the left side of the body, for each of the 4 conditions), it was possible to classify above chance which participants are patients with MS and which are controls. The AUC for each of the models of the separate conditions was: “Free walk” = 0.71 ± 0.17;” Click-Walk” = 0.71 ± 0.14; “0-back” = 0.76 ± 0.15; “2-back” = 0.76 ± 0.14. Figure 1 shows the ROC curves of these models.

The 15 most relevant variables are presented in a relative importance table (Table 1).

**Table 1:** Values of the 15 most relevant variables for distinguishing groups of patients with multiple sclerosis (EDSS 0 to 3.5) and controls without the diagnosis using the average.

Variable	Control (*N* = 19)	MS (*N* = 19)
L_Mean_Right_Knee FO		
Average (SD)	43.7 (3.45)	39.5 (4.25)
Median (Min, Max)	44.1 (38.2, 51.2)	39.7 (28.4, 45.7)
C_Mean_Right_TrunkRotationRange		
Average (SD)	7.10 (2.92)	9.53 (2.86)
Median (Min, Max)	7.41 (0, 11.9)	9.14 (6.28, 17.3)
F_Mean_Right_Pelvic rotation in stance		
Average (SD)	46.0 (19.0)	57.6 (13.9)
Median (Min, Max)	46.7 (17.3, 83.7)	59.3 (34.7, 80.0)
F_Mean_Left_TrunkRotationFO		
Average (SD)	2.44 (2.76)	-2.45 (8.53)
Median (Min, Max)	2.43 (−1.59, 7.14)	0.0226 (−29.1, 6.29)
F_Mean_Left _Peak dorsi		
Average (SD)	43.6 (4.33)	46.9 (3.74)
Median (Min, Max)	44.3 (31.1, 48.2)	48.1 (39.8, 51.8)
C_Mean_Right_PelvicObliquityRange		
Average (SD)	11.4 (2.34)	9.63 (2.42)
Median (Min, Max)	11.7 (6.56, 15.8)	9.10 (5.94, 13.6)
A_Mean_Right_Knee terminal stance		
Average (SD)	38.1 (17.3)	50.0 (11.4)
Median (Min, Max)	43.0 (1.25, 62.3)	53.5 (20.8, 65.0)
L_Mean_Left_DorsiPlanFlexTMN		
Average (SD)	64.3 (1.42)	62.1 (6.79)
Median (Min, Max)	64.1 (61.1. 67.1)	64.6 (46.2, 69.5)
L_Mean_Right_Dorsi in stance		
Average (SD)	13.7 (2.61)	12.1 (3.35)
Median (Min, Max)	13.5 (8.88, 18.1)	12.7 (3.90, 17.8)
C_Mean_Right_Dorsi FO		
Average (SD)	−20.8 (5.66)	−16.3 (5.64)
Median (Min, Max)	−20.4 (−31.0, −11.3)	−17.1 (−26.3, −5.37)
L_Mean_Left_Knee FO		
Average (SD)	42.6 (4.89)	39.5 (4.01)
Median (Min, Max)	42.1 (33.6, 49.9)	39.9 (28.0, 46.2)
A_Mean_Right_TrunkRotationRange		
Average (SD)	7.28 (2.97)	9.19 (2.17)
Median (Min, Max)	7.15 (0, 12.4)	8.73 (6.48, 13.6)
A_Mean_Left_TrunkRotationRange		
Average (SD)	7.25 (2.75)	9.16 (2.27)
Median (Min, Max)	6.90 (0, 12.5)	8.47 (6.60, 13.7)
L_Mean_Left_OppositeFootContact		
Average (SD)	50.1 (0.640)	50.4 (0.840)
Median (Min, Max)	50.1 (49.0, 51.2)	50.5 (49.0, 52.1)
C_Mean_Left_Pelvic Rotation in stance		
Average (SD)	50.8 (9.34)	58.6 (12.0)
Median (Min, Max)	49.7 (33.2, 71.8)	58.6 (38.7, 85.8)
C_Mean_Right_TrunkObliquityRange		
Average (SD)	3.07 (1.22)	3.96 (1.46)
Median (Min, Max)	3.01 (0, 5.32)	3.84 (2.12, 7.32)

C—click walk; L—2-back; A—0-back; F—free walk; FO—foot off (the moment the foot leaves the ground); TMX—maximum peak; AVG—range of movement; IC—initial contact; MNS—minimum peak; TMN—terminal phase of medium support; TMNAFP—position or alignment of the foot and ankle during the terminal phase of mid-stance; PKD—peak dorsiflexion (maximum point of ankle dorsiflexion during the gait cycle).

### Classification using average and standard deviation

Using the average and standard deviation variables the best model achieved AUC = 0.94 ± 0.09 (average ± SD). This means that using 1,200 gait kinematic variables (300 in each of the 4 conditions) we were able to classify above chance who is a patient with Multiple Sclerosis and who is a control participant.

The AUC for each of the models of the separate conditions was: “Free walk” = 0.94 ± 0.09;” Click-Walk” = 0.89 ± 0.11; “0-back” = 0.83 ± 0.14; “2-back” = 0.81 ± 0.15. Table 2 shows the relative importance of the 20 most relevant variables.

**Table 2:** Values of the 15 most relevant variables for distinguishing groups of patients with multiple sclerosis (EDSS 0 to 3.5) and controls without the diagnosis using the mean and standard deviation.

Variable	Control (*N* = 19)	MS (*N* = 19)
F_SD_Left_FootProgression		
Average (SD)	1.34 (0.418)	2.10 (0.586)
Median (Min, Max)	1.22 (0.676, 2.15)	2.01 (1.08, 3.47)
F_SD_Right_PelvicRotation		
Average (SD)	0.916 (0.277)	1.42 (0.400)
Median (Min, Max)	0.956 (0.374, 1.32)	1.32 (0.826, 2.16)
F_SD_Right_PelvicRotationMNS		
Average (SD)	1.21 (0.326)	1.88 (0.500)
Median (Min, Max)	1.23 (0.546, 1.63)	1.76 (0.800, 3.02)
A_SD_Right_HipFlexExtIC		
Average (SD)	1.18 (0.349)	1.77 (0.554)
Median (Min, Max)	1.13 (0.529, 1.91)	1.64 (0.471, 2.87)
F_SD_Right_Cadence		
Average (SD)	2.04 (0.714)	3.21 (1.09)
Median (Min, Max)	1.92 (0.931, 3.49)	3.25 (1.82, 6.10)
F_SD_Right_StrideTime		
Average (SD)	0.0196 (0.00743)	0.0328 (0.0118)
Median (Min, Max)	0.0171 (0.00878, 0.0355)	0.0300 (0.0157, 0.0552)
C_SD_Left_StrideLength		
Average (SD)	0.0238 (0.00856)	0.0343 (0.00943)
Median (Min, Max)	0.0232 (0.0105, 0.0411)	0.0315 (0.0221, 0.0568)
C_SD_Right_PelvicRotation		
Average (SD)	1.58 (0.456)	2.17 (0.556)
Median (Min, Max)	1.58 (0.721, 2.35)	2.30 (0.905, 2.96)
C_SD_Right_HipFlexRange		
Average (SD)	1.41 (0.462)	1.99 (0.488)
Median (Min, Max)	1.52 (0.625. 2.19)	2.06 (1.15, 2.67)
F_SD_Left_PelvicRotationMAX		
Average (SD)	1.08 (0.367)	1.64 (0.490)
Median (Min, Max)	1.03 (0.466, 1.72)	1.53 (1.01, 2.72)
L_SD_Right_TrunkObliquityRange		
Average (SD)	0.569 (0.283)	1.44 (0.515)
Median (Min, Max)	0.530 (0, 1.07)	0.952 (0.350, 1.25)
C_SD_Right_HipRotationMIN		
Average (SD)	1.01 (0.309)	1.44 (0.515)
Median (Min, Max)	0.950 (0.563, 1.90)	1.30 (0.794, 2.60)
L_Mean_Right_KneeFLexExtFO		
Average (SD)	43.7 (3.45)	39.5 (4.25)
Median (Min, Max)	44.1 (38.2, 51.2)	39.7 (28.4, 45.7)
A_SD_Left_PelvicTiltAVG		
Average (SD)	0.550 (0.177)	0.837 (0.260)
Median (Min, Max)	0.482 (0.202, 0.942)	0.900 (0.456, 1.34)
F_SD_Right_PelvicRotationMIN		
Average (SD)	1.20 (0.325)	1.73 (0.493)
Median (Min, Max)	1.23 (0.672, 1.63)	1.73 (0.791, 2.52)
C_SD_Right_FootProgressionAVG		
Average (SD)	1.64 (0.782)	2.56 (0.830)
Median (Min, Max)	1.68 (0.414, 3.63)	2.36 (1.56, 5.31)
C_SD_Right_KneeFlexExtPKSW		
Average (SD)	1.10 (0.429)	1.77 (0.720)
Median (Min, Max)	1.01 (0.439, 1.90)	1.54 (0.966, 3.33)
F_Mean_Right_PelvicRotationTMX		
Average (SD)	46.0 (19.0)	57.6 (13.9)
Median (Min, Max)	46.7 (17.3, 83.7)	59.3 (34.7, 80.0)
F_SD_Left_PelvicRotationAVGST		
Average (SD)	0.929 (0.289)	1.40 (0.503)
Median (Min, Max)	0.898 (0.529, 1.57)	1.50 (0.579, 2.15)
A_Mean_Right_KneeFlexExtTMNAFP		
Average (SD)	38.1 (17.3)	50.0 (11.4)
Median (Min, Max)	43.0 (1.25, 62.3)	53.5 (20.8, 65.0)

C—click walk; L—0-back; A—2-back; F—free walk; FO—foot off (the moment the foot leaves the ground); TMX—maximum peak; AVG—range of movement; IC—initial contact; MNS—minimum peak; TMN—terminal phase of medium support; TMNAFP—position or alignment of the foot and ankle during the terminal phase of mid-stance; PKD—peak dorsiflexion (maximum point of ankle dorsiflexion during the gait cycle).

### Classification using standard deviation

Using the standard deviation variables the best model achieved AUC = 0.95 ± 0.09 (average ± SD). This means that using 600 gait kinematic variables in each of the 4 conditions we were able to classify above chance who is a patient with Multiple Sclerosis and who is a control participant.

The AUC for each of the models of the separate conditions was: “Free walk” = 0.95 ± 0.09;” Click-Walk” = 0.89 ± 0.11; “0-back” = 0.83 ± 0.12; “2-back” = 0.78 ± 0.14. Table 3 the relative importance of the 20 most relevant variables.

**Table 3:** Values of the 20 most relevant variables for distinguishing groups of patients with multiple sclerosis (EDSS 0 to 3.5) and controls without the diagnosis using the standard deviation.

Variable	Control (*N* = 19)	MS (*N* = 19)
F_SD_Left_FootProgression		
Average (SD)	1.34 (0.418)	2.10 (0.586)
Median (Min, Max)	1.22 (0.676, 2.15)	2.01 (1.08, 3.47)
F_SD_Right_PelvicRotationAVGST		
Average (SD)	0.916 (0.277)	1.42 (0.400)
Median (Min, Max)	0.956 (0.374, 1.32)	1.32 (0.826, 2.16)
F_SD_Right_PelvicRotationMNS		
Average (SD)	1.21 (0.326)	1.88 (0.500)
Median (Min, Max)	1.23 (0.546, 1.63)	1.76 (0.800, 3.02)
A_SD_Right_HipIC		
Average (SD)	1.18 (0.349)	1.77 (0.554)
Median (Min, Max)	1.13 (0.529, 1.91)	1.64 (0.471, 2.87)
C_SD_Left_StrideLength		
Average (SD)	0.0238 (0.00856)	0.0343 (0.00943)
Median (Min, Max)	0.0232 (0.0105, 0.0411)	0.0315 (0.0221, 0.0568)
F_SD_Right_StrideTime		
Average (SD)	0.0196 (0.00743)	0.0328 (0.0118)
Median (Min, Max)	0.0171 (0.00878, 0.0355)	0.0300 (0.0157, 0.0552)
F_SD_Right_Cadence		
Average (SD)	2.04 (0.714)	3.21 (1.09)
Median (Min, Max)	1.92 (0.931, 3.49)	3.25 (1.82, 6.10)
C_SD_Right_HipFlexExtRange		
Average (SD)	1.41 (0.462)	1.99 (0.488)
Median (Min, Max)	1.52 (0.625, 2.19)	2.06 (1.15, 2.67)
C_SD_Right_HipRotationMin		
Average (SD)	1.01 (0.309)	1.44 (0.515)
Median (Min, Max)	0.950 (0.563, 1.90)	1.30 (0.794, 2.60)
L_SD_Right_TrunkObliquityRange		
Average (SD)	0.569 (0.283)	0.853 (0.257)
Median (Min, Max)	0.530 (0, 1.07)	0.952 (0.350, 1.25)
C_SD_Right_PelvicRotationRange		
Average (SD)	1.58 (0.456)	2.17 (0.556)
Median (Min, Max)	1.58 (0.721, 2.35)	2.30 (0.905, 2.96)
A_SD_Left_PelvicTil		
Average (SD)	0.550 (0.177)	0.837 (0.260)
Median (Min, Max)	0.482 (0.202, 0.942)	0.900 (0.456, 1.34)
F_SD_Left_PelvicRotationMAX		
Average (SD)	1.08 (0.367)	1.64 (0.490)
Median (Min, Max)	1.03 (0.466, 1.72)	1.53 (1.01, 2.72)
F_SD_Right_PelvicRotationMIN		
Average (SD)	1.20 (0.325)	1.73 (0.493)
Median (Min, Max)	1.23 (0.672, 1.63)	1.73 (0.791, 2.52)
C_SD_Right_KneeFlexExtPKSW		
Average (SD)	1.10 (0.429)	1.77 (0.720)
Median (Min, Max)	1.01 (0.439, 1.90)	1.54 (0.966, 3.33)
A_SD_Left_SingleSupport		
Average (SD)	0.0128 (0.00411)	0.0181 (0.00672)
Median (Min, Max)	0.0132 (0.00653, 0.0212)	0.0156 (0.0106, 0.0376)
F_SD_Left_PelvicRotationAVGST		
Average (SD)	0.929 (0.289)	1.40 (0.503)
Median (Min, Max)	0.898 (0.529, 1.57)	1.50 (0.579, 2.15)
C_SD_Left_TrunkObliquityRange		
Average (SD)	0.594 (0.191)	1.01 (0.502)
Median (Min, Max)	0.609 (0, 0.914)	0.929 (0.444, 2.81)
F_SD_Right_StePTime		
Average (SD)	0.0152 (0.00444)	0.0234 (0.00868)
Median (Min, Max)	0.0145 (0.00694, 0.0245)	0.0217 (0.0113, 0.0454)
C_SD_Right_FootProgressionAVG		
Average (SD)	1.64 (0.782)	2.56 (0.830)
Median (Min, Max)	1.68 (0.414, 3.63)	2.36 (1.56, 5.31)

C—click walk; L—0-back; A—2-back; F—free walk; FO—foot off (the moment the foot leaves the ground); TMX—maximum peak; AVG—range of movement; IC—initial contact; MNS—minimum peak; TMN—terminal phase of medium support; TMNAFP—position or alignment of the foot and ankle during the terminal phase of mid-stance; PKD—peak dorsiflexion (maximum point of ankle dorsiflexion during the gait cycle).

As summarized in Figure 1, the Elastic Net model demonstrates notable improvements when incorporating standard deviation of kinematic variables, either alone or in combination with average values. This visual representation underscores the superior performance achieved with variability measures compared to using only average values across all task conditions (Overall, Free Walk, Click-Walk, 0-Back, and 2-Back). The visualization shows that models incorporating standard deviation, either alone or in combination with mean values, generally achieve significantly higher classification using AUC compared to those relying solely on average kinematic variables. This highlights the importance of variability in gait parameters for distinguishing between individuals with early-stage Multiple Sclerosis and healthy controls.

## Discussion

The aim of this study was to verify whether it is possible to classify patients in the early stages of multiple sclerosis and healthy controls into different groups, using angular gait data, both during normal gait (free walk) and during dual-task (click-walk, 0-back and 2-back).

When considering only the average gait data for classification analysis, the best model achieved AUC = 0.77 ± 0.21 in cross-validation, suggesting an ability to differentiate between groups above the level of chance.

When the average and standard deviation of kinematic variables were considered, there was a notable improvement in the model’s performance, reaching an AUC of 0.94 ± 0.09. These results indicate that the incorporation of gait data measured by standard deviation improves the model, enhancing its ability to distinguish between patients with MS multiple sclerosis and healthy individuals. We even carried out an analysis using only the standard deviation data, but the results were very similar to those of standard deviation with average.

Recent research indicates that variables related to walking, especially in dual-task conditions, may reveal early motor impairments in patients with MS. The study highlights the importance of using kinematic analysis to identify specific walking problems (Shanahan et al., 2018). Our study suggests that machine-learning algorithms can detect changes in walking patterns, even in the early stages of multiple sclerosis, during walking.

The specific kinematic variables identified as most relevant for classification in our models offer valuable insights into the subtle yet distinct gait alterations in early-stage MS. For instance, the increased variability (standard deviation) observed across numerous parameters—including foot progression, pelvic rotation, stride time, and cadence (Tables 2, 3)—is a critical indicator of compromised motor control and reduced gait stability in neurological conditions (Socie et al., 2013; Demnitz et al., 2016). This heightened variability suggests a less automatic and more cognitively effortful walking pattern, even in individuals with low EDSS scores, as their central nervous system struggles to maintain consistent movement patterns (Woollacott and Shumway-Cook, 2002). The automaticity of the impaired free walking may be masked by more self controlled movements during dual task which probably requires cognitive control for both the task and the walking. Furthermore, changes in average angular ranges such as altered knee angles at foot off or in terminal stance, and increased trunk and pelvic rotation ranges (Table 1), likely represent compensatory strategies for underlying weakness or spasticity, common in MS, affecting shock absorption, propulsion, and overall gait efficiency (Martin et al., 2006; Cameron and Lord, 2010). These findings underscore that while overt disability may be minimal, quantifiable kinematic deviations are present, serving as early markers of the disease’s impact on mobility and potentially contributing to an increased risk of falls and reduced functional independence over time (Shanahan et al., 2018). The ability of machine learning to discern these specific and often subtle patterns reinforces its potential as a sensitive tool for early detection and objective assessment in clinical settings.

The efficacy of machine learning in discerning subtle gait patterns, as suggested in our study, is further supported by recent advancements in the field. For instance, new methodologies in human gait pattern recognition, particularly those with applications in clinical settings, underscore the power of computational approaches in identifying nuanced differences not readily apparent through conventional assessments (Xu et al., 2024). Similarly, studies employing machine learning to differentiate gait patterns even in high-performance contexts, such as between runners with varying mileage, reinforce the robustness and sensitivity of these techniques in capturing subtle biomechanical distinctions (Xu et al., 2022). While the general approach of utilizing and combining statistical features like average and standard deviation for gait analysis is present in the broader field of human movement analysis, as exemplified by works such as Pan et al. (2017) on gait recognition using sensor data, our specific contribution lies in systematically demonstrating its efficacy and, more importantly, highlighting the superior discriminatory power of variability metrics (standard deviation) over average values in the challenging context of early-stage Multiple Sclerosis (MS) with low disability (EDSS scores 0–3.5). This insight, suggesting that machine learning models using gait variability may detect subtle gait impairments in early MS, was not applied in previous studies within this specific clinical population. The Elastic Net model, chosen for its robust feature selection capabilities in high-dimensional datasets, allowed us to identify these relevant kinematic variability parameters that contribute most effectively to the classification. These external validations highlight the potential of our Elastic Net model not only for classifying early-stage MS but also for contributing to a broader understanding of pathological gait characteristics, thereby offering more comprehensive and effective insights into neurological conditions.

The study by Özgür et al. (2024) emphasizes that gait changes in patients with MS may involve a reduction in speed, cadence and step length factors associated with an increased risk of falls and decreased independence. The study indicates that the comparison between patients and controls shows an AUC of 0.84 ± 0.12. This suggests that this method is useful in detecting minor motor problems, which aligns with the belief that movement pattern analysis can help detect early signs of changes in walking patterns.

A study by Ramírez and Gutiérrez (2021) emphasize how supervised learning methods such as Elastic Net improve the accuracy of analysis and provide important information about the progression of MS. This helps with early detection and interventions.

In previous research, the results indicate similarities and noticeable differences. Research conducted by Rooney et al. (2020) points to the worsening of dual-task gait impairment in people with MS due to the increase in cognitive demands that exacerbate motor deficits.

Furthermore, the effectiveness of the model is improved by incorporating regularization methods such as Elastic Net, which present practical benefits for clinical environments. Recent research indicates that Elastic Net stands out in the selection of variables in scenarios with many dimensions and variable correlations, surpassing the conventional dimension reduction technique, such as Principal Component Analysis (Hastie et al., 2009).

To improve the understanding of gait changes related to MS, future research should adopt a multidimensional approach that integrates kinematic data with clinical assessments. Detecting changes in motor skills, such as difficulty walking quickly, is vital to improving treatments and slowing down the progression of disabilities (Shanahan et al., 2018).

While our study provides valuable insights into the classification of early-stage MS patients using gait kinematics and machine learning, it is important to acknowledge some limitations. Firstly, the relatively small sample size (19 participants per group) represents a convenience sample. Despite rigorous matching for demographic variables, this size precluded a formal power analysis to ascertain definitive statistical power, meaning our promising findings should be considered preliminary and exploratory. Furthermore, our study specifically focused on individuals with early-stage MS and mild disability (EDSS scores between 0 and 3.5). While this specific focus allowed us to detect subtle gait deviations in a population where clinical assessment is often challenging, the generalizability of these findings to patients with more advanced stages of MS or higher levels of disability requires further investigation. Secondly, the cross-sectional nature of the study prevents the assessment of longitudinal changes in gait patterns or the long-term predictive power of our model. A further consideration is that while we meticulously controlled for disease activity by including only patients with stable MS for at least 6 months, we did not explicitly account for disease duration or the specific types and regimens of treatments used by the MS participants. These factors are known to influence disease progression and symptomatology, including gait; however, their direct inclusion as covariates was challenging due to their exclusive presence in the MS group and the inherent heterogeneity of medication types within our sample. Lastly, although the Elastic Net model proved highly effective for our specific objective of classification and feature identification in this high-dimensional dataset, a direct comparative analysis with other machine learning algorithms (e.g., Support Vector Machines, Random Forests) was beyond the scope of this preliminary report. Addressing these points, future research is crucial. This includes conducting larger, multi-center, and longitudinal studies to enhance generalizability and validate findings across a broader spectrum of early MS presentations, including those with varying levels of disability. Such studies should also investigate the specific impact of disease duration and different pharmacological interventions on kinematic gait variables, and comprehensively compare the performance of various machine learning models to further optimize classification accuracy and generalize findings.

In conclusion, this study suggests that using angular gait analysis with machine learning methods is a successful way to categorize individuals with early-stage multiple sclerosis and those who are otherwise healthy. The research also provides a substantial contribution to the existing literature by introducing a viable method for detecting early signs of motor changes related to multiple sclerosis.

## Data Availability

The raw data supporting the conclusions of this article will be made available by the authors, without undue reservation.
