# Supplementary material for: Classification of patients with early-stage multiple sclerosis and healthy controls using kinematic analysis during a dual-task
Source: Front Artif Intell. 2025 Oct 21;8:1660801. doi: 10.3389/frai.2025.1660801 (PMC12582952; doi:10.3389/frai.2025.1660801)
Supplement: Supplementary file 1 [file Data_Sheet_1.pdf]

Supplementary Material

S1. Experimental Setup and Data Acquisition

Gait data were acquired using a high-precision **Vicon® motion capture system**, which comprised 10 infrared cameras strategically positioned to generate three-dimensional kinematic data of the lower limbs. Spherical reflective markers, made of polystyrene, were affixed to the participants' skin according to the established **Plug-in Gait protocol** to define specific anatomical landmarks (as illustrated in Figure 1 of the main manuscript).

- **Calibration Procedures:** Prior to each data collection session, standard Vicon system calibration procedures were meticulously performed. This included both static and dynamic calibrations to accurately define the laboratory's coordinate system and optimize marker tracking precision. These rigorous calibration steps ensured the reliability and accuracy of the captured kinematic data, which is critical for detecting subtle gait deviations.

- **Data Types Collected:** The system concurrently captured both spatiotemporal parameters (such as speed, cadence, stride length, and single/double support time) and angular kinematic data. The angular data focused on the ankle, knee, hip, and trunk joints across various events of the gait cycle. For the purpose of this specific study, **only the angular kinematic data were utilized** for subsequent analysis.

All experimental sessions and data acquisitions were conducted at the Einstein Movement Study Laboratory (LEME) within the Hospital Israelita Albert Einstein (HIAE).

S2. Experimental Task Protocol

The experimental protocol was designed to assess gait performance under both free-walking and dual-task conditions. The cognitive load was introduced through an n-back task, which evaluates working memory by requiring participants to recall an audio-presented number from 'n' positions ago. The experimental design followed a block paradigm (as illustrated in Figure 2 of the main manuscript) with the following specific instructions for each condition:

1. **Free Walk:**
   - **Instruction:** Participants were instructed to walk at a comfortable, self-selected speed for a minimum duration of 1 minute.
   - **Purpose:** This phase served to collect baseline gait parameters without any cognitive interference.
2. **Dual-Task Conditions:**
   - **Overall Setup:** While walking at a comfortable speed, participants simultaneously performed one of three distinct cognitive tasks. Each dual-task block lasted 20 seconds and was interspersed with 15-second pauses to allow for baseline collection between tasks.

- 0-back Block: Upon hearing a sequence of numbers from 0 to 9, the participant was required to press a response button whenever the number '0' was heard.
- **2-back Block:** Upon hearing a sequence of numbers from 1 to 9, the participant was required to press a response button whenever the current number was identical to the number presented two positions prior in the sequence.
- **Click-Walk Block:** The participant's task was to press a response button immediately upon hearing the auditory command 'click'.
- *Note:* Participants consistently walked at a comfortable speed throughout all tasks. The response time to the cognitive stimuli was not a evaluated variable in this study.

S3. Kinematic Data Processing and Variable Derivation

From the comprehensive gait data collected, the analysis was specifically focused on angular kinematic variables. These variables included angular movements of the ankle, knee, hip, and trunk across different phases and events of the gait cycle. For each relevant body segment and gait event, two primary kinematic variables were derived and utilized in the classification models:

- **Mean (AVG):** The average angular value.
- **Range of Motion (ADM/ROM):** The total range of angular movement.

These derived angular data points formed the basis for the subsequent machine learning classification.

S4. Statistical Analysis and Machine Learning Model Details

Descriptive Statistics: Initial data characterization involved the use of descriptive statistics to identify absolute and relative angular frequencies. Means, medians, and standard deviations were also reported for all relevant variables.

Classification Model: An **Elastic Net regression model** was employed to assess its ability to classify between MS patients and healthy controls based on the collected kinematic data.

Model Optimization and Validation:

- The model parameters were optimized using a Machine Learning methodology.
- Due to the limited sample size, a separate test set was not created. Instead, model validation and parameter tuning were rigorously performed using a **5-fold cross-validation with 5 repetitions**.
- The primary performance metric used was the **average classification accuracy** across the cross-validation folds.
- During parameter tuning, the alpha parameter (balancing Ridge and Lasso regularization) ranged from 0 to 1 (in 0.01 intervals), and the lambda parameter (penalty weight) ranged from 0 to 50 (in 0.05 intervals).

Elastic Net Specifics: Elastic Net is a powerful regularization technique widely applied in linear regression models, particularly beneficial in contexts characterized by a large number of variables and high multicollinearity among them. This technique intelligently combines the strengths of both Least Absolute Shrinkage and Selection Operator (Lasso) and Ridge regression to enhance predictive accuracy and achieve robust variable selection.

- The alpha parameter in Elastic Net controls the balance between Lasso and Ridge regularization:
  - When alpha = 1, the model behaves purely as **Lasso regression**, which applies a penalty based on the sum of the absolute values of the coefficients. This can drive less relevant variable coefficients to exactly zero, thus performing automatic variable selection and producing a sparser model.
  - When alpha = 0, the model behaves purely as **Ridge regression**, applying a penalty on the sum of the squared coefficients. Ridge is particularly effective in handling multicollinearity by distributing weights more evenly among correlated variables, leading to increased robustness.
  - **Intermediate alpha values (between 0 and 1)** allow the model to leverage the advantages of both methods, enabling a more dynamic adjustment to the data structure and inherent variable correlations. This approach promotes the selection of a subset of informative variables for prediction while penalizing both the absolute and squared values of coefficients, resulting in a sparse model that effectively manages multicollinearity.

For implementation, the Elastic Net model was applied using specialized packages within the R statistical software environment, such as glmnet. This framework facilitated the adjustment of penalized models with flexibility in defining regularization levels and balancing the influences of Lasso and Ridge, thereby improving the model's ability to generalize to new data and identify key variables even in high-dimensional datasets.

S5. RMarkdown analysis code and packages version

The analysis code is provided below. The code was executed using R version 4.3.2 (2023-10-31 ucrt) -- "Eye Holes", and run under RStudio version 2023.12.0 Build 369 - "Ocean Storm" Release (33206f75, 2023-12-14) for windows. The packages attached to the session were:

attached base packages:

- parallel
- stats
- graphics
- grDevices
- utils
- datasets
- methods
- base

other attached packages (and their version)

- gridExtra_2.3

- pROC_1.18.5
- table1 (Version 1.4.3)
- doParallel (Version 1.0.17)
- iterators (Version 1.0.14)
- foreach (Version 1.5.2)
- glmnet (Version 4.1-8)
- Matrix (Version 1.6-4)
- reshape2 (Version 1.4.4)
- ggpubr (Version 0.6.0)
- lemon (Version 0.4.7)
- caret (Version 6.0-94)
- lattice (Version 0.21-9)
- lubridate (Version 1.9.3)
- forcats (Version 1.0.0)
- stringr (Version 1.5.1)
- dplyr (Version 1.1.4)
- purrr (Version 1.0.2)
- readr (Version 2.1.4)
- tidyr (Version 1.3.0)
- tibble (Version 3.2.1)
- ggplot2 (Version 3.4.4)
- tidyverse (Version 2.0.0)
- here (Version 1.0.1)

Analysis code:

````
---
title: "Multiple sclerosis gait"
author: "Raymundo Neto and Paulo Bazán"
date: "19/09/2025 (dd/mm/yyyy)"
output: html_document
---

<style>
body {
text-align: justify;
font-family: "Helvetica"}

h1.title {
font-family: "Helvetica"
}
</style>

<p style='text-align: justify;'><font size="4"> Multiple Sclerosis project lead by Elisa Kozasa.


```{r, echo=FALSE, warning=FALSE, message=FALSE}
library(here)
library(tidyverse)
library(caret)
library(lemon)
````
````

~~~
knit_print.data.frame <- lemon_print
library(ggpubr)
library(reshape2)
library(glmnet)
library(doParallel)
library(parallel)
library(table1)
library(pROC) #for ROC estimation of variable importance (currently not used)
library(dplyr) #for plotting
library(gridExtra)
library(grid)
```

```{r, echo=FALSE, warning=FALSE, message=FALSE}
# Setting folder paths
curr_dir = here()
#data_dir = paste(curr_dir,"../1_Original_Data/1_Original_Data/",sep ="/")
#clean_data_dir = paste(curr_dir,"../2_Analysis/",sep ="/")
#output_dir = paste(curr_dir,"../5_Output/",sep ="/")

# Load data
dataset = read.csv(file = paste(curr_dir,"Dados_Cinematicos_V2.csv", sep = "/"),header = T
RUE, sep = ",",dec = ".",fileEncoding="UTF-8-BOM")

names(dataset)[which(names(dataset) == "Grupo")]="Group"

# Change data type of some columns
dataset = dataset %>% mutate_at(c("Group"), as.factor)

#Selecting Mean and/or SD variables
#Here we select just the name of the F condition and use these names to select the corresp
onding variables in each condition
var_names=dataset %>% select(starts_with(c("F_Mean"))) %>% names
var_names_SD=gsub("Mean","SD",var_names)
#var_names_SD=dataset %>% select(starts_with(c("F_SD"))) %>% names
var_names_Mean_SD=c(var_names,var_names_SD)

dataset_filtered=dataset %>% select(ends_with(c(substr(var_names,start = 3,stop = 100),"G
roup")))
dataset_filtered_SD=dataset %>% select(ends_with(c(substr(var_names_SD,start = 3,stop
= 100),"Group")))
dataset_filtered_Mean_SD=dataset %>% select(ends_with(c(substr(var_names_Mean_SD,s
tart = 3,stop = 100),"Group")))

Fset=dataset_filtered %>% select(starts_with(c("F_Mean","Group")))
Cset=dataset_filtered %>% select(starts_with(c("C_Mean","Group")))
Aset=dataset_filtered %>% select(starts_with(c("A_Mean","Group")))
Lset=dataset_filtered %>% select(starts_with(c("L_Mean","Group")))

Fset_SD=dataset_filtered_SD %>% select(starts_with(c("F_SD","Group")))
Cset_SD=dataset_filtered_SD %>% select(starts_with(c("C_SD","Group")))
Aset_SD=dataset_filtered_SD %>% select(starts_with(c("A_SD","Group")))
Lset_SD=dataset_filtered_SD %>% select(starts_with(c("L_SD","Group")))

Fset_Mean_SD=dataset_filtered_Mean_SD %>% select(starts_with(c("F_","Group")))
~~~

```
Cset_Mean_SD=dataset_filtered_Mean_SD %>% select(starts_with(c("C_","Group")))
Aset_Mean_SD=dataset_filtered_Mean_SD %>% select(starts_with(c("A_","Group")))
Lset_Mean_SD=dataset_filtered_Mean_SD %>% select(starts_with(c("L_","Group")))

# choosing alpha:
alpha_value = seq(0,1,length.out = 101) #Defines the weight between Lasso and Ridge regr
essions -> alpha = 1 means Lasso, and alpha = 0 means Ridge

lambda_value = seq(0.05,50,length.out = 1000) #Applied to both ridge and lasso. The equati
on used buy elastic net is: lambda *(alpha*Lasso + (1-alpha)*Ridge) -> ridge shrinks variable
s, Lasso eliminates them.

#Prepare parallel processing:
ncores=detectCores()

cl <- makePSOCKcluster(ncores)
registerDoParallel(cl)

## Setting parameters used in all models:

# Simpler version if you only care about Accuracy
customSummary <- function(data, lev = NULL, model = NULL) {
  # Calculate twoClassSummary metrics
  twoClass <- twoClassSummary(data, lev, model)

  # Calculate accuracy only
  acc <- mean(data$pred == data$obs)

  # Combine results
  c(twoClass, Accuracy = acc)
}

# Add this custom summary function to your code
customSummary <- function(data, lev = NULL, model = NULL) {
  # Calculate twoClassSummary metrics
  twoClass <- twoClassSummary(data, lev, model)

  # Calculate accuracy
  acc <- defaultSummary(data, lev, model)["Accuracy"]

  # Combine results
  c(twoClass, Accuracy = acc)
}

# Then update your trainControl
myControl = trainControl(method = "repeatedcv",
                         number = 5,
                         repeats = 5,
                         summaryFunction = customSummary,  # to get ACC and ROC
                         classProbs = TRUE,
                         savePredictions = "final",
                         verboseIter = FALSE)


# Set up tuning parameters
```

````
myTuning = expand.grid(alpha = alpha_value, lambda = lambda_value)

# Set up preprocessing
myPreproc = c("knnImpute") #Automatically scales the data (https://topepo.github.io/caret/pre-processing.html#pp)

# Function to extract best result from cross-validation
get_best_result = function(caret_fit) {
  best = which(rownames(caret_fit$results) == rownames(caret_fit$bestTune))
  best_result = caret_fit$results[best, ]
  rownames(best_result) = NULL
  best_result
}

#variable to store the ROC plots
ROCplots = array(list(), 12)
#Initializing Model counter
modelcounter=1

```
````

Analysis with 75 Variables from each side (V2)

Classification analysis using Mean variables

Classification of patients and controls with all conditions together.

We trained the elastic net model to classify participants as being from the Multiple sclerosis or control group using the means of several gait kinematic variables.

The model was trained using a 5-fold cross validation with 5 repetitions and parameter tuning was performed with alpha ranging from 0 to 1 and lambda ranging from 0.05 to 50. The performance metric is classification average accuracy across cross-validation folds. Because the sample size is small, we did not create a separate test set, only validation sets for the cross-validation procedure.

````
```{r, echo=FALSE, warning=FALSE, message=FALSE}
# random number seed
set.seed(1245)

# Fit model to training data
model = train(Group ~ .,
              data = dataset_filtered,
              tuneGrid = myTuning,
              trControl = myControl,
              preProcess = myPreproc,
              method = "glmnet",
              family = "binomial")

results=get_best_result(model)
cv_acc = results$Accuracy.Accuracy
cv_acc_sd = results$Accuracy.AccuracySD
cv_alpha = results$alpha
cv_lambda = results$lambda
if (sum(model$finalModel$lambda < results[[2]])==0) {
````

~~~~
  used_coef=model$finalModel$df[length(model$finalModel$df)]
}else{
  used_coef=model$finalModel$df[which(model$finalModel$lambda < results[[2]])[[1]]]
}
cv_auc <- results$ROC  # If you have twoClassSummary enabled
cv_auc_sd <- results$ROCSD

```
~~~~

Cross-validation accuracy = `r round(cv_acc,2)` $\pm$ `r round(cv_acc_sd,2)`(mean $\pm$ SD). This means that using `r dim(dataset_filtered)[2]-1` gait kinematic variables (`r (dim(dataset_filtered)[2]-1)/4` in each of the 4 conditions) we are able to classify above chance who is a patient with Multiple Sclerosis and who is a control participant.

This is also suggested by the cross-validated AUC = `r round(cv_auc,2)` $\pm$ `r round(cv_auc_sd,2)` and by the ROC curve.

~~~~
```{r, echo=FALSE, warning=FALSE, message=FALSE, fig.align='center'}
### Generating Threshold-Averaged ROC curve
# Get predictions for best hyperparameters
best_preds <- model$pred %>%
  filter(alpha == cv_alpha & lambda == cv_lambda)

# Create common FPR grid for threshold averaging
fpr_grid <- seq(0, 1, length.out = 100)

# Calculate TPR at each FPR point for each fold
tpr_matrix <- matrix(NA, nrow = length(fpr_grid), ncol = length(unique(best_preds$Resample
)))
folds <- unique(best_preds$Resample)

for(i in seq_along(folds)) {
  fold_data <- best_preds %>% filter(Resample == folds[i])
  roc_fold <- roc(fold_data$obs, fold_data$EM)

  # Get TPR at each FPR point using interpolation
  tpr_matrix[, i] <- approx(x = 1 - roc_fold$specificities,
                        y = roc_fold$sensitivities,
                        xout = fpr_grid,
                        method = "linear",
                        rule = 2)$y
}

# Calculate mean and SD of TPR across folds at each FPR point
threshold_avg_roc <- data.frame(
  FPR = fpr_grid,
  mean_TPR = rowMeans(tpr_matrix, na.rm = TRUE),
  sd_TPR = apply(tpr_matrix, 1, sd, na.rm = TRUE)
) %>%
  mutate(
    upper_TPR = pmin(mean_TPR + sd_TPR, 1),
    lower_TPR = pmax(mean_TPR - sd_TPR, 0)
  )

# Plot threshold-averaged ROC curve
~~~~

````
threshold_roc_plot <- ggplot(threshold_avg_roc, aes(x = FPR, y = mean_TPR)) +
  geom_ribbon(aes(ymin = lower_TPR, ymax = upper_TPR),
              alpha = 0.2, fill = "blue", color = NA) +
  geom_line(color = "blue", linewidth = 1.5) +
  geom_abline(slope = 1, intercept = 0, linetype = "dashed", color = "red", linewidth = 1) +
  labs(title = paste("Threshold-Averaged Cross-Validated ROC Curve\n(AUC =", round(cv_a
uc, 3), ")"),
       x = "False Positive Rate (1 - Specificity)",
       y = "True Positive Rate (Sensitivity)") +
  theme_minimal() +
  theme(plot.title = element_text(hjust = 0.5, size = 14),
        axis.title = element_text(size = 12),
        axis.text = element_text(size = 10)) +
  coord_equal(ratio = 1) +
  scale_x_continuous(limits = c(0, 1), expand = c(0, 0)) +
  scale_y_continuous(limits = c(0, 1), expand = c(0, 0))

print(threshold_roc_plot)

#storing the current plot
ROCplots[[modelcounter]] = threshold_roc_plot
```


### Importance of variables for the estimated model

The best model was achieved with alpha = `r cv_alpha`, and lambda = `r cv_lambda`

Below is a plot of the relative importance of the `r ifelse(used_coef <=20, used_coef,20)` mo
st relevant variables.

```{r, echo=FALSE, warning=FALSE, message=FALSE, fig.align='center'}
temp1=varImp(model)
temp2=sort(as.matrix(temp1$importance),decreasing = TRUE,index.return = TRUE)
if (used_coef<=20){
  plot(temp1,top = used_coef)
} else {
  plot(temp1,top = 20)
}

```


#### Table of the most important variables for the estimated model

```{r, echo=FALSE, warning=FALSE, message=FALSE, fig.align='center'}
if (used_coef<=20){
  topvarnames=formula(paste("~ ", paste(row.names(temp1$importance)[temp2$ix[1:used_c
oef]], collapse= "+")," | Group"))
} else {
  topvarnames=formula(paste("~ ", paste(row.names(temp1$importance)[temp2$ix[1:20]], co
llapse= "+")," | Group"))
}
table1(topvarnames,dataset_filtered,overall=FALSE)
```
````
````

````
```{r, echo=FALSE, warning=FALSE, message=FALSE}
#Optional to save the results (all R space) of each model
save.image(file = paste("Model_",modelcounter,".RData",sep = ""))
modelcounter = modelcounter + 1 #even if the above line is commented, keep the counter update active for the ROCplots
```
````

Classification in each condition separately

After the analysis with all conditions, we decided to explore each experimental condition separately. Legend of the experimental conditions: F = Free Walk (single task experiment);C = Click-Walk (Walk in the dual task n-back experiment) A = 0-back (during dual task n-back experiment); and L = 2-back (during the dual-task n-back experiment).

Condition Free Walk during single task experiment

````
```{r, echo=FALSE, warning=FALSE, message=FALSE}
# random number seed
set.seed(1245)

# Fit model to training data
model_reduced = train(Group ~ .,
            data = Fset,
            tuneGrid = myTuning,
            trControl = myControl,
            preProcess = myPreproc,
            method = "glmnet",
            family = "binomial")

results_reduced=get_best_result(model_reduced)
cv_acc = results_reduced$Accuracy.Accuracy
cv_acc_sd = results_reduced$Accuracy.AccuracySD
cv_alpha = results_reduced$alpha
cv_lambda = results_reduced$lambda
if (sum(model_reduced$finalModel$lambda < results[[2]])==0) {
  used_coef=model_reduced$finalModel$df[length(model_reduced$finalModel$df)]
}else{
  used_coef=model_reduced$finalModel$df[which(model_reduced$finalModel$lambda < results_reduced[[2]])[[1]]]
}
cv_auc <- results_reduced$ROC  # If you have twoClassSummary enabled
cv_auc_sd <- results_reduced$ROCSD
```
````

Using data from free walk condition only, the performance of the model seemed a little bit worse: Accuracy = `r round(cv_acc, 2)` $\pm$ `r round(cv_acc_sd,2)`.

This is also suggested by the cross-validated AUC = `r round(cv_auc,2)` $\pm$ `r round(cv_auc_sd,2)` and by the ROC curve.

````
```{r, echo=FALSE, warning=FALSE, message=FALSE, fig.align='center'}
### Generating Threshold-Averaged ROC curve
# Get predictions for best hyperparameters
best_preds <- model_reduced$pred %>%
````
````

```
  filter(alpha == cv_alpha & lambda == cv_lambda)

# Create common FPR grid for threshold averaging
fpr_grid <- seq(0, 1, length.out = 100)

# Calculate TPR at each FPR point for each fold
tpr_matrix <- matrix(NA, nrow = length(fpr_grid), ncol = length(unique(best_preds$Resample
)))
folds <- unique(best_preds$Resample)

for(i in seq_along(folds)) {
  fold_data <- best_preds %>% filter(Resample == folds[i])
  roc_fold <- roc(fold_data$obs, fold_data$EM)

  # Get TPR at each FPR point using interpolation
  tpr_matrix[, i] <- approx(x = 1 - roc_fold$specificities,
                          y = roc_fold$sensitivities,
                          xout = fpr_grid,
                          method = "linear",
                          rule = 2)$y
}

# Calculate mean and SD of TPR across folds at each FPR point
threshold_avg_roc <- data.frame(
  FPR = fpr_grid,
  mean_TPR = rowMeans(tpr_matrix, na.rm = TRUE),
  sd_TPR = apply(tpr_matrix, 1, sd, na.rm = TRUE)
) %>%
  mutate(
    upper_TPR = pmin(mean_TPR + sd_TPR, 1),
    lower_TPR = pmax(mean_TPR - sd_TPR, 0)
  )

# Plot threshold-averaged ROC curve
threshold_roc_plot <- ggplot(threshold_avg_roc, aes(x = FPR, y = mean_TPR)) +
  geom_ribbon(aes(ymin = lower_TPR, ymax = upper_TPR),
            alpha = 0.2, fill = "blue", color = NA) +
  geom_line(color = "blue", linewidth = 1.5) +
  geom_abline(slope = 1, intercept = 0, linetype = "dashed", color = "red", linewidth = 1) +
  labs(title = paste("Threshold-Averaged Cross-Validated ROC Curve\n(AUC =", round(cv_a
uc, 3), ")"),
      x = "False Positive Rate (1 - Specificity)",
      y = "True Positive Rate (Sensitivity)") +
  theme_minimal() +
  theme(plot.title = element_text(hjust = 0.5, size = 14),
       axis.title = element_text(size = 12),
       axis.text = element_text(size = 10)) +
  coord_equal(ratio = 1) +
  scale_x_continuous(limits = c(0, 1), expand = c(0, 0)) +
  scale_y_continuous(limits = c(0, 1), expand = c(0, 0))

print(threshold_roc_plot)

#storing the current plot
ROCplots[[modelcounter]] = threshold_roc_plot
```

```

#### Importance of variables for the estimated model

The best model was achieved with alpha = `r cv_alpha`, and lambda = `r cv_lambda`.

Below is a plot of the relative importance of the `r ifelse(used_coef <=20, used_coef,20)` mo
st relevant variables.

```{r, echo=FALSE, warning=FALSE, message=FALSE, fig.align='center'}
temp1=varImp(model_reduced)
temp2=sort(as.matrix(temp1$importance),decreasing = TRUE,index.return = TRUE)
if (used_coef<=20){
  plot(temp1,top = used_coef)
} else {
  plot(temp1,top = 20)
}

```

Table of the most important variables for the estimated model

```{r, echo=FALSE, warning=FALSE, message=FALSE, fig.align='center'}
if (used_coef<=20){
  topvarnames=formula(paste("~ ", paste(row.names(temp1$importance)[temp2$ix[1:used_c
oef]], collapse= "+")," | Group"))
} else {
  topvarnames=formula(paste("~ ", paste(row.names(temp1$importance)[temp2$ix[1:20]], co
llapse= "+")," | Group"))
}
table1(topvarnames,dataset_filtered,overall=FALSE)
```

```{r, echo=FALSE, warning=FALSE, message=FALSE}
#Optional to save the results (all R space) of each model
save.image(file = paste("Model_",modelcounter,".RData",sep = ""))
modelcounter = modelcounter + 1 #even if the above line is commented, keep the counter u
pdate active for the ROCplots
```

Condition Walk during the dual task experiment

```{r, echo=FALSE, warning=FALSE, message=FALSE}
# random number seed
set.seed(1245)

# Fit model to training data
model_reduced = train(Group ~ .,
            data = Cset,
            tuneGrid = myTuning,
            trControl = myControl,
            preProcess = myPreproc,
            method = "glmnet",
            family = "binomial")

results_reduced=get_best_result(model_reduced)
cv_acc = results_reduced$Accuracy.Accuracy
cv_acc_sd = results_reduced$Accuracy.AccuracySD
cv_alpha = results_reduced$alpha
cv_lambda = results_reduced$lambda
if (sum(model_reduced$finalModel$lambda < results[[2]])==0) {
  used_coef=model_reduced$finalModel$df[length(model_reduced$finalModel$df)]
}else{
  used_coef=model_reduced$finalModel$df[which(model_reduced$finalModel$lambda < results_reduced[[2]])[[1]]]
}
cv_auc <- results_reduced$ROC  # If you have twoClassSummary enabled
cv_auc_sd <- results_reduced$ROCSD
```

Using data from walk condition of the dual task experiment, the performance of the model was: Accuracy = `r round(cv_acc, 2)` $\pm$ `r round(cv_acc_sd,2)`.

Cross-validated AUC = `r round(cv_auc,2)` $\pm$ `r round(cv_auc_sd,2)`.

The ROC curve is presented below.

```{r, echo=FALSE, warning=FALSE, message=FALSE, fig.align='center'}
### Generating Threshold-Averaged ROC curve
# Get predictions for best hyperparameters
best_preds <- model_reduced$pred %>%
  filter(alpha == cv_alpha & lambda == cv_lambda)

# Create common FPR grid for threshold averaging
fpr_grid <- seq(0, 1, length.out = 100)

# Calculate TPR at each FPR point for each fold
tpr_matrix <- matrix(NA, nrow = length(fpr_grid), ncol = length(unique(best_preds$Resample)))
folds <- unique(best_preds$Resample)

for(i in seq_along(folds)) {
  fold_data <- best_preds %>% filter(Resample == folds[i])
  roc_fold <- roc(fold_data$obs, fold_data$EM)

  # Get TPR at each FPR point using interpolation
  tpr_matrix[, i] <- approx(x = 1 - roc_fold$specificities,
                            y = roc_fold$sensitivities,
                            xout = fpr_grid,
                            method = "linear",
                            rule = 2)$y
}

# Calculate mean and SD of TPR across folds at each FPR point
threshold_avg_roc <- data.frame(
  FPR = fpr_grid,
  mean_TPR = rowMeans(tpr_matrix, na.rm = TRUE),
  sd_TPR = apply(tpr_matrix, 1, sd, na.rm = TRUE)
) %>%
  mutate(

    upper_TPR = pmin(mean_TPR + sd_TPR, 1),
    lower_TPR = pmax(mean_TPR - sd_TPR, 0)
  )

# Plot threshold-averaged ROC curve
threshold_roc_plot <- ggplot(threshold_avg_roc, aes(x = FPR, y = mean_TPR)) +
  geom_ribbon(aes(ymin = lower_TPR, ymax = upper_TPR),
              alpha = 0.2, fill = "blue", color = NA) +
  geom_line(color = "blue", linewidth = 1.5) +
  geom_abline(slope = 1, intercept = 0, linetype = "dashed", color = "red", linewidth = 1) +
  labs(title = paste("Threshold-Averaged Cross-Validated ROC Curve\n(AUC =", round(cv_a
uc, 3), ")"),
       x = "False Positive Rate (1 - Specificity)",
       y = "True Positive Rate (Sensitivity)") +
  theme_minimal() +
  theme(plot.title = element_text(hjust = 0.5, size = 14),
        axis.title = element_text(size = 12),
        axis.text = element_text(size = 10)) +
  coord_equal(ratio = 1) +
  scale_x_continuous(limits = c(0, 1), expand = c(0, 0)) +
  scale_y_continuous(limits = c(0, 1), expand = c(0, 0))

print(threshold_roc_plot)

#storing the current plot
ROCplots[[modelcounter]] = threshold_roc_plot
```

Importance of variables for the estimated model

The best model was achieved with alpha = `r cv_alpha`, and lambda = `r cv_lambda`.

Below is a plot of the relative importance of the `r ifelse(used_coef <=20, used_coef,20)` mo
st relevant variables.

```{r, echo=FALSE, warning=FALSE, message=FALSE, fig.align='center'}
temp1=varImp(model_reduced)
temp2=sort(as.matrix(temp1$importance),decreasing = TRUE,index.return = TRUE)
if (used_coef<=20){
  plot(temp1,top = used_coef)
} else {
  plot(temp1,top = 20)
}

```

Table of the most important variables for the estimated model

```{r, echo=FALSE, warning=FALSE, message=FALSE, fig.align='center'}
if (used_coef<=20){
  topvarnames=formula(paste("~ ", paste(row.names(temp1$importance)[temp2$ix[1:used_c
oef]], collapse= "+")," | Group"))
} else {
  topvarnames=formula(paste("~ ", paste(row.names(temp1$importance)[temp2$ix[1:20]], co
llapse= "+")," | Group"))

}
table1(topvarnames,dataset_filtered,overall=FALSE)
```

```{r, echo=FALSE, warning=FALSE, message=FALSE}
#Optional to save the results (all R space) of each model
save.image(file = paste("Model_",modelcounter,".RData",sep = ""))
modelcounter = modelcounter + 1 #even if the above line is commented, keep the counter u
pdate active for the ROCplots
```

Condition Find during the dual task experiment

```{r, echo=FALSE, warning=FALSE, message=FALSE}
# random number seed
set.seed(1245)

# Fit model to training data
model_reduced = train(Group ~ .,
            data = Aset,
            tuneGrid = myTuning,
            trControl = myControl,
            preProcess = myPreproc,
            method = "glmnet",
            family = "binomial")

results_reduced=get_best_result(model_reduced)
cv_acc = results_reduced$Accuracy.Accuracy
cv_acc_sd = results_reduced$Accuracy.AccuracySD
cv_alpha = results_reduced$alpha
cv_lambda = results_reduced$lambda
if (sum(model_reduced$finalModel$lambda < results[[2]])==0) {
  used_coef=model_reduced$finalModel$df[length(model_reduced$finalModel$df)]
}else{
  used_coef=model_reduced$finalModel$df[which(model_reduced$finalModel$lambda < res
ults_reduced[[2]])[[1]]]
}
cv_auc <- results_reduced$ROC  # If you have twoClassSummary enabled
cv_auc_sd <- results_reduced$ROCSD
```

Using data from Find condition of the dual task experiment, the performance of the model wa
s: Accuracy = `r round(cv_acc, 2)` $\pm$ `r round(cv_acc_sd,2)`.

Cross-validated AUC = `r round(cv_auc,2)` $\pm$ `r round(cv_auc_sd,2)`.

The ROC curve is presented below.

```{r, echo=FALSE, warning=FALSE, message=FALSE, fig.align='center'}
### Generating Threshold-Averaged ROC curve
# Get predictions for best hyperparameters
best_preds <- model_reduced$pred %>%
  filter(alpha == cv_alpha & lambda == cv_lambda)

# Create common FPR grid for threshold averaging

```
fpr_grid <- seq(0, 1, length.out = 100)

# Calculate TPR at each FPR point for each fold
tpr_matrix <- matrix(NA, nrow = length(fpr_grid), ncol = length(unique(best_preds$Resample
)))
folds <- unique(best_preds$Resample)

for(i in seq_along(folds)) {
  fold_data <- best_preds %>% filter(Resample == folds[i])
  roc_fold <- roc(fold_data$obs, fold_data$EM)

  # Get TPR at each FPR point using interpolation
  tpr_matrix[, i] <- approx(x = 1 - roc_fold$specificities,
                            y = roc_fold$sensitivities,
                            xout = fpr_grid,
                            method = "linear",
                            rule = 2)$y
}

# Calculate mean and SD of TPR across folds at each FPR point
threshold_avg_roc <- data.frame(
  FPR = fpr_grid,
  mean_TPR = rowMeans(tpr_matrix, na.rm = TRUE),
  sd_TPR = apply(tpr_matrix, 1, sd, na.rm = TRUE)
) %>%
  mutate(
    upper_TPR = pmin(mean_TPR + sd_TPR, 1),
    lower_TPR = pmax(mean_TPR - sd_TPR, 0)
  )

# Plot threshold-averaged ROC curve
threshold_roc_plot <- ggplot(threshold_avg_roc, aes(x = FPR, y = mean_TPR)) +
  geom_ribbon(aes(ymin = lower_TPR, ymax = upper_TPR),
              alpha = 0.2, fill = "blue", color = NA) +
  geom_line(color = "blue", linewidth = 1.5) +
  geom_abline(slope = 1, intercept = 0, linetype = "dashed", color = "red", linewidth = 1) +
  labs(title = paste("Threshold-Averaged Cross-Validated ROC Curve\n(AUC =", round(cv_a
uc, 3), ")"),
       x = "False Positive Rate (1 - Specificity)",
       y = "True Positive Rate (Sensitivity)") +
  theme_minimal() +
  theme(plot.title = element_text(hjust = 0.5, size = 14),
        axis.title = element_text(size = 12),
        axis.text = element_text(size = 10)) +
  coord_equal(ratio = 1) +
  scale_x_continuous(limits = c(0, 1), expand = c(0, 0)) +
  scale_y_continuous(limits = c(0, 1), expand = c(0, 0))

print(threshold_roc_plot)

#storing the current plot
ROCplots[[modelcounter]] = threshold_roc_plot
```

Importance of variables for the estimated model

The best model was achieved with alpha = `r cv_alpha`, and lambda = `r cv_lambda`.

Below is a plot of the relative importance of the `r ifelse(used_coef <=20, used_coef,20)` most relevant variables.

```{r, echo=FALSE, warning=FALSE, message=FALSE, fig.align='center'}
temp1=varImp(model_reduced)
temp2=sort(as.matrix(temp1$importance),decreasing = TRUE,index.return = TRUE)
if (used_coef<=20){
  plot(temp1,top = used_coef)
} else {
  plot(temp1,top = 20)
}

```

Table of the most important variables for the estimated model

```{r, echo=FALSE, warning=FALSE, message=FALSE, fig.align='center'}
if (used_coef<=20){
  topvarnames=formula(paste("~ ", paste(row.names(temp1$importance)[temp2$ix[1:used_coef]], collapse= "+")," | Group"))
} else {
  topvarnames=formula(paste("~ ", paste(row.names(temp1$importance)[temp2$ix[1:20]], collapse= "+")," | Group"))
}
table1(topvarnames,dataset_filtered,overall=FALSE)
```

```{r, echo=FALSE, warning=FALSE, message=FALSE}
#Optional to save the results (all R space) of each model
save.image(file = paste("Model_",modelcounter,".RData",sep = ""))
modelcounter = modelcounter + 1 #even if the above line is commented, keep the counter update active for the ROCplots
```

Condition Remember during the dual-task experiment

```{r, echo=FALSE, warning=FALSE, message=FALSE}
# random number seed
set.seed(1245)

# Fit model to training data
model_reduced = train(Group ~ .,
            data = Lset,
            tuneGrid = myTuning,
            trControl = myControl,
            preProcess = myPreproc,
            method = "glmnet",
            family = "binomial")

results_reduced=get_best_result(model_reduced)
cv_acc = results_reduced$Accuracy.Accuracy
cv_acc_sd = results_reduced$Accuracy.AccuracySD
```

```
cv_alpha = results_reduced$alpha
cv_lambda = results_reduced$lambda
if (sum(model_reduced$finalModel$lambda < results[[2]])==0) {
  used_coef=model_reduced$finalModel$df[length(model_reduced$finalModel$df)]
}else{
  used_coef=model_reduced$finalModel$df[which(model_reduced$finalModel$lambda < res
ults_reduced[[2]])[[1]]]
}
cv_auc <- results_reduced$ROC  # If you have twoClassSummary enabled
cv_auc_sd <- results_reduced$ROCSD
```

Using data from Remember condition of the dual task experiment, the performance of the model improved was: Accuracy = `r round(cv_acc, 2)` $\pm$ `r round(cv_acc_sd,2)`.

Cross-validated AUC = `r round(cv_auc,2)` $\pm$ `r round(cv_auc_sd,2)`.

The ROC curve is presented below.

```{r, echo=FALSE, warning=FALSE, message=FALSE, fig.align='center'}
### Generating Threshold-Averaged ROC curve
# Get predictions for best hyperparameters
best_preds <- model_reduced$pred %>%
  filter(alpha == cv_alpha & lambda == cv_lambda)

# Create common FPR grid for threshold averaging
fpr_grid <- seq(0, 1, length.out = 100)

# Calculate TPR at each FPR point for each fold
tpr_matrix <- matrix(NA, nrow = length(fpr_grid), ncol = length(unique(best_preds$Resample
)))
folds <- unique(best_preds$Resample)

for(i in seq_along(folds)) {
  fold_data <- best_preds %>% filter(Resample == folds[i])
  roc_fold <- roc(fold_data$obs, fold_data$EM)

  # Get TPR at each FPR point using interpolation
  tpr_matrix[, i] <- approx(x = 1 - roc_fold$specificities,
                         y = roc_fold$sensitivities,
                         xout = fpr_grid,
                         method = "linear",
                         rule = 2)$y
}

# Calculate mean and SD of TPR across folds at each FPR point
threshold_avg_roc <- data.frame(
  FPR = fpr_grid,
  mean_TPR = rowMeans(tpr_matrix, na.rm = TRUE),
  sd_TPR = apply(tpr_matrix, 1, sd, na.rm = TRUE)
) %>%
  mutate(
    upper_TPR = pmin(mean_TPR + sd_TPR, 1),
    lower_TPR = pmax(mean_TPR - sd_TPR, 0)
  )
```

# Plot threshold-averaged ROC curve
threshold_roc_plot <- ggplot(threshold_avg_roc, aes(x = FPR, y = mean_TPR)) +
  geom_ribbon(aes(ymin = lower_TPR, ymax = upper_TPR),
              alpha = 0.2, fill = "blue", color = NA) +
  geom_line(color = "blue", linewidth = 1.5) +
  geom_abline(slope = 1, intercept = 0, linetype = "dashed", color = "red", linewidth = 1) +
  labs(title = paste("Threshold-Averaged Cross-Validated ROC Curve\n(AUC =", round(cv_auc, 3), ")"),
       x = "False Positive Rate (1 - Specificity)",
       y = "True Positive Rate (Sensitivity)") +
  theme_minimal() +
  theme(plot.title = element_text(hjust = 0.5, size = 14),
        axis.title = element_text(size = 12),
        axis.text = element_text(size = 10)) +
  coord_equal(ratio = 1) +
  scale_x_continuous(limits = c(0, 1), expand = c(0, 0)) +
  scale_y_continuous(limits = c(0, 1), expand = c(0, 0))

print(threshold_roc_plot)

#storing the current plot
ROCplots[[modelcounter]] = threshold_roc_plot
```

#### Importance of variables for the estimated model

The best model was achieved with alpha = `r cv_alpha`, and lambda = `r cv_lambda`.

Below is a plot of the relative importance of the `r ifelse(used_coef <=20, used_coef,20)` most relevant variables.

```{r, echo=FALSE, warning=FALSE, message=FALSE, fig.align='center'}
temp1=varImp(model_reduced)
temp2=sort(as.matrix(temp1$importance),decreasing = TRUE,index.return = TRUE)
if (used_coef<=20){
  plot(temp1,top = used_coef)
} else {
  plot(temp1,top = 20)
}

```

Table of the most important variables for the estimated model

```{r, echo=FALSE, warning=FALSE, message=FALSE, fig.align='center'}
if (used_coef<=20){
  topvarnames=formula(paste("~ ", paste(row.names(temp1$importance)[temp2$ix[1:used_coef]], collapse= "+")," | Group"))
} else {
  topvarnames=formula(paste("~ ", paste(row.names(temp1$importance)[temp2$ix[1:20]], collapse= "+")," | Group"))
}
table1(topvarnames,dataset_filtered,overall=FALSE)
```
```

```{r, echo=FALSE, warning=FALSE, message=FALSE}
#Optional to save the results (all R space) of each model
save.image(file = paste("Model_",modelcounter,".RData",sep = ""))
modelcounter = modelcounter + 1 #even if the above line is commented, keep the counter u
pdate active for the ROCplots
```

Classification analysis using Mean and SD variables

As the classification was not as good as expected, in a post hoc analysis we decided to inclu
de the SD variables to check if they would increase the model accuracy.

Classification of patients and controls with all conditions together.

We trained the elastic net model to classify participants as being from the Multiple sclerosis
or control group using the means of several gait kinematic variables.

The model was trained using a 5-fold cross validation with 5 repetitions and parameter tunin
g was performed with alpha ranging from 0 to 1 and lambda ranging from 0.05 to 50. The per
formance metric is classification average accuracy across cross-validation folds. Because th
e sample size is small, we did not create a separate test set, only validation sets for the cros
s-validation procedure.

```{r, echo=FALSE, warning=FALSE, message=FALSE}
# random number seed
set.seed(1245)

# Fit model to training data
model = train(Group ~ .,
            data = dataset_filtered_Mean_SD,
            tuneGrid = myTuning,
            trControl = myControl,
            preProcess = myPreproc,
            method = "glmnet",
            family = "binomial")

results=get_best_result(model)
cv_acc = results$Accuracy.Accuracy
cv_acc_sd = results$Accuracy.AccuracySD
cv_alpha = results$alpha
cv_lambda = results$lambda
cv_auc <- results$ROC  # If you have twoClassSummary enabled
cv_auc_sd <- results$ROCSD
if (sum(model$finalModel$lambda < results[[2]])==0) {
  used_coef=model$finalModel$df[length(model$finalModel$df)]
}else{
  used_coef=model$finalModel$df[which(model$finalModel$lambda < results[[2]])[[1]]]
}

```

Cross-validation accuracy = `r round(cv_acc,2)` $\pm$ `r round(cv_acc_sd,2)`(mean $\pm$
SD). This means that using `r dim(dataset_filtered_Mean_SD)[2]-1` gait kinematic variables (

`r (dim(dataset_filtered_Mean_SD)[2]-1)/4` in each of the 4 conditions) we are able to classif
y above chance who is a patient with Multiple Sclerosis and who is a control participant.

Cross-validated AUC = `r round(cv_auc,2)` $\pm$ `r round(cv_auc_sd,2)`.

The ROC curve is presented below.

```{r, echo=FALSE, warning=FALSE, message=FALSE, fig.align='center'}
### Generating Threshold-Averaged ROC curve
# Get predictions for best hyperparameters
best_preds <- model$pred %>%
  filter(alpha == cv_alpha & lambda == cv_lambda)

# Create common FPR grid for threshold averaging
fpr_grid <- seq(0, 1, length.out = 100)

# Calculate TPR at each FPR point for each fold
tpr_matrix <- matrix(NA, nrow = length(fpr_grid), ncol = length(unique(best_preds$Resample
)))
folds <- unique(best_preds$Resample)

for(i in seq_along(folds)) {
  fold_data <- best_preds %>% filter(Resample == folds[i])
  roc_fold <- roc(fold_data$obs, fold_data$EM)

  # Get TPR at each FPR point using interpolation
  tpr_matrix[, i] <- approx(x = 1 - roc_fold$specificities,
                          y = roc_fold$sensitivities,
                          xout = fpr_grid,
                          method = "linear",
                          rule = 2)$y
}

# Calculate mean and SD of TPR across folds at each FPR point
threshold_avg_roc <- data.frame(
  FPR = fpr_grid,
  mean_TPR = rowMeans(tpr_matrix, na.rm = TRUE),
  sd_TPR = apply(tpr_matrix, 1, sd, na.rm = TRUE)
) %>%
  mutate(
    upper_TPR = pmin(mean_TPR + sd_TPR, 1),
    lower_TPR = pmax(mean_TPR - sd_TPR, 0)
  )

# Plot threshold-averaged ROC curve
threshold_roc_plot <- ggplot(threshold_avg_roc, aes(x = FPR, y = mean_TPR)) +
  geom_ribbon(aes(ymin = lower_TPR, ymax = upper_TPR),
            alpha = 0.2, fill = "blue", color = NA) +
  geom_line(color = "blue", linewidth = 1.5) +
  geom_abline(slope = 1, intercept = 0, linetype = "dashed", color = "red", linewidth = 1) +
  labs(title = paste("Threshold-Averaged Cross-Validated ROC Curve\n(AUC =", round(cv_a
uc, 3), ")"),
       x = "False Positive Rate (1 - Specificity)",
       y = "True Positive Rate (Sensitivity)") +
  theme_minimal() +
```

  theme(plot.title = element_text(hjust = 0.5, size = 14),
        axis.title = element_text(size = 12),
        axis.text = element_text(size = 10)) +
  coord_equal(ratio = 1) +
  scale_x_continuous(limits = c(0, 1), expand = c(0, 0)) +
  scale_y_continuous(limits = c(0, 1), expand = c(0, 0))

print(threshold_roc_plot)

#storing the current plot
ROCplots[[modelcounter]] = threshold_roc_plot
```

### Importance of variables for the estimated model

The best model was achieved with alpha = `r cv_alpha`, and lambda = `r cv_lambda`. This s
mall lambda indicates that the model used is close to a traditional model (without Lasso or Ri
dge corrections).

Below is a plot of the relative importance of the `r ifelse(used_coef <=20, used_coef,20)` mo
st relevant variables.

```{r, echo=FALSE, warning=FALSE, message=FALSE, fig.align='center'}
temp1=varImp(model)
temp2=sort(as.matrix(temp1$importance),decreasing = TRUE,index.return = TRUE)
if (used_coef<=20){
  plot(temp1,top = used_coef)
} else {
  plot(temp1,top = 20)
}

```

Table of the most important variables for the estimated model

```{r, echo=FALSE, warning=FALSE, message=FALSE, fig.align='center'}
if (used_coef<=20){
  topvarnames=formula(paste("~ ", paste(row.names(temp1$importance)[temp2$ix[1:used_c
oef]], collapse= "+")," | Group"))
} else {
  topvarnames=formula(paste("~ ", paste(row.names(temp1$importance)[temp2$ix[1:20]], co
llapse= "+")," | Group"))
}
table1(topvarnames,dataset_filtered_Mean_SD,overall=FALSE)
```

```{r, echo=FALSE, warning=FALSE, message=FALSE}
#Optional to save the results (all R space) of each model
save.image(file = paste("Model_",modelcounter,".RData",sep = ""))
modelcounter = modelcounter + 1 #even if the above line is commented, keep the counter u
pdate active for the ROCplots
```

Classification in each condition separately
```

After the analysis with all conditions, we decided to explore each experimental condition separately. Legend of the experimental conditions: F = Free Walk (single task experiment);C = Click-Walk (Walk in the dual task n-back experiment) A = 0-back (during dual task n-back experiment); and L = 2-back (during the dual-task n-back experiment).

Condition Free Walk during single task experiment

```{r, echo=FALSE, warning=FALSE, message=FALSE}
# random number seed
set.seed(1245)

# Fit model to training data
model_reduced = train(Group ~ .,
            data = Fset_Mean_SD,
            tuneGrid = myTuning,
            trControl = myControl,
            preProcess = myPreproc,
            method = "glmnet",
            family = "binomial")

results_reduced=get_best_result(model_reduced)
cv_acc = results_reduced$Accuracy.Accuracy
cv_acc_sd = results_reduced$Accuracy.AccuracySD
cv_alpha = results_reduced$alpha
cv_lambda = results_reduced$lambda
if (sum(model_reduced$finalModel$lambda < results[[2]])==0) {
  used_coef=model_reduced$finalModel$df[length(model_reduced$finalModel$df)]
}else{
  used_coef=model_reduced$finalModel$df[which(model_reduced$finalModel$lambda < results_reduced[[2]])[[1]]]
}
cv_auc <- results_reduced$ROC  # If you have twoClassSummary enabled
cv_auc_sd <- results_reduced$ROCSD
```

Using data from free walk condition only, the performance of the model was still very good: Accuracy = `r round(cv_acc, 2)` $\pm$ `r round(cv_acc_sd,2)`.

Cross-validated AUC = `r round(cv_auc,2)` $\pm$ `r round(cv_auc_sd,2)`.

The ROC curve is presented below.

```{r, echo=FALSE, warning=FALSE, message=FALSE, fig.align='center'}
### Generating Threshold-Averaged ROC curve
# Get predictions for best hyperparameters
best_preds <- model_reduced$pred %>%
  filter(alpha == cv_alpha & lambda == cv_lambda)

# Create common FPR grid for threshold averaging
fpr_grid <- seq(0, 1, length.out = 100)

# Calculate TPR at each FPR point for each fold
tpr_matrix <- matrix(NA, nrow = length(fpr_grid), ncol = length(unique(best_preds$Resample)))
```

```
folds <- unique(best_preds$Resample)

for(i in seq_along(folds)) {
  fold_data <- best_preds %>% filter(Resample == folds[i])
  roc_fold <- roc(fold_data$obs, fold_data$EM)

  # Get TPR at each FPR point using interpolation
  tpr_matrix[, i] <- approx(x = 1 - roc_fold$specificities,
                            y = roc_fold$sensitivities,
                            xout = fpr_grid,
                            method = "linear",
                            rule = 2)$y
}

# Calculate mean and SD of TPR across folds at each FPR point
threshold_avg_roc <- data.frame(
  FPR = fpr_grid,
  mean_TPR = rowMeans(tpr_matrix, na.rm = TRUE),
  sd_TPR = apply(tpr_matrix, 1, sd, na.rm = TRUE)
) %>%
  mutate(
    upper_TPR = pmin(mean_TPR + sd_TPR, 1),
    lower_TPR = pmax(mean_TPR - sd_TPR, 0)
  )

# Plot threshold-averaged ROC curve
threshold_roc_plot <- ggplot(threshold_avg_roc, aes(x = FPR, y = mean_TPR)) +
  geom_ribbon(aes(ymin = lower_TPR, ymax = upper_TPR),
              alpha = 0.2, fill = "blue", color = NA) +
  geom_line(color = "blue", linewidth = 1.5) +
  geom_abline(slope = 1, intercept = 0, linetype = "dashed", color = "red", linewidth = 1) +
  labs(title = paste("Threshold-Averaged Cross-Validated ROC Curve\n(AUC =", round(cv_auc, 3), ")"),
       x = "False Positive Rate (1 - Specificity)",
       y = "True Positive Rate (Sensitivity)") +
  theme_minimal() +
  theme(plot.title = element_text(hjust = 0.5, size = 14),
        axis.title = element_text(size = 12),
        axis.text = element_text(size = 10)) +
  coord_equal(ratio = 1) +
  scale_x_continuous(limits = c(0, 1), expand = c(0, 0)) +
  scale_y_continuous(limits = c(0, 1), expand = c(0, 0))

print(threshold_roc_plot)

#storing the current plot
ROCplots[[modelcounter]] = threshold_roc_plot
```

Importance of variables for the estimated model

The best model was achieved with alpha = `r cv_alpha`, and lambda = `r cv_lambda`.

Below is a plot of the relative importance of the `r ifelse(used_coef <=20, used_coef,20)` most relevant variables.

```{r, echo=FALSE, warning=FALSE, message=FALSE, fig.align='center'}
temp1=varImp(model_reduced)
temp2=sort(as.matrix(temp1$importance),decreasing = TRUE,index.return = TRUE)
if (used_coef<=20){
  plot(temp1,top = used_coef)
} else {
  plot(temp1,top = 20)
}

```

Table of the most important variables for the estimated model

```{r, echo=FALSE, warning=FALSE, message=FALSE, fig.align='center'}
if (used_coef<=20){
  topvarnames=formula(paste("~ ", paste(row.names(temp1$importance)[temp2$ix[1:used_coef]], collapse= "+")," | Group"))
} else {
  topvarnames=formula(paste("~ ", paste(row.names(temp1$importance)[temp2$ix[1:20]], collapse= "+")," | Group"))
}
table1(topvarnames,dataset_filtered_Mean_SD,overall=FALSE)
```

```{r, echo=FALSE, warning=FALSE, message=FALSE}
#Optional to save the results (all R space) of each model
save.image(file = paste("Model_",modelcounter,".RData",sep = ""))
modelcounter = modelcounter + 1 #even if the above line is commented, keep the counter update active for the ROCplots
```

Condition Walk during the dual task experiment

```{r, echo=FALSE, warning=FALSE, message=FALSE}
# random number seed
set.seed(1245)

# Fit model to training data
model_reduced = train(Group ~ .,
            data = Cset_Mean_SD,
            tuneGrid = myTuning,
            trControl = myControl,
            preProcess = myPreproc,
            method = "glmnet",
            family = "binomial")

results_reduced=get_best_result(model_reduced)
cv_acc = results_reduced$Accuracy.Accuracy
cv_acc_sd = results_reduced$Accuracy.AccuracySD
cv_alpha = results_reduced$alpha
cv_lambda = results_reduced$lambda
cv_auc <- results_reduced$ROC  # If you have twoClassSummary enabled
cv_auc_sd <- results_reduced$ROCSD
```

````
if (sum(model_reduced$finalModel$lambda < results[[2]])==0) {
  used_coef=model_reduced$finalModel$df[length(model_reduced$finalModel$df)]
}else{
  used_coef=model_reduced$finalModel$df[which(model_reduced$finalModel$lambda < results_reduced[[2]])[[1]]]
}

```
````

Using data from walk condition of the dual task experiment, the performance of the model was: Accuracy = `r round(cv_acc, 2)` $\pm$ `r round(cv_acc_sd,2)`.

Cross-validated AUC = `r round(cv_auc,2)` $\pm$ `r round(cv_auc_sd,2)`.

The ROC curve is presented below.

````
```{r, echo=FALSE, warning=FALSE, message=FALSE, fig.align='center'}
### Generating Threshold-Averaged ROC curve
# Get predictions for best hyperparameters
best_preds <- model_reduced$pred %>%
  filter(alpha == cv_alpha & lambda == cv_lambda)

# Create common FPR grid for threshold averaging
fpr_grid <- seq(0, 1, length.out = 100)

# Calculate TPR at each FPR point for each fold
tpr_matrix <- matrix(NA, nrow = length(fpr_grid), ncol = length(unique(best_preds$Resample)))
folds <- unique(best_preds$Resample)

for(i in seq_along(folds)) {
  fold_data <- best_preds %>% filter(Resample == folds[i])
  roc_fold <- roc(fold_data$obs, fold_data$EM)

  # Get TPR at each FPR point using interpolation
  tpr_matrix[, i] <- approx(x = 1 - roc_fold$specificities,
                          y = roc_fold$sensitivities,
                          xout = fpr_grid,
                          method = "linear",
                          rule = 2)$y
}

# Calculate mean and SD of TPR across folds at each FPR point
threshold_avg_roc <- data.frame(
  FPR = fpr_grid,
  mean_TPR = rowMeans(tpr_matrix, na.rm = TRUE),
  sd_TPR = apply(tpr_matrix, 1, sd, na.rm = TRUE)
) %>%
  mutate(
    upper_TPR = pmin(mean_TPR + sd_TPR, 1),
    lower_TPR = pmax(mean_TPR - sd_TPR, 0)
  )

# Plot threshold-averaged ROC curve
threshold_roc_plot <- ggplot(threshold_avg_roc, aes(x = FPR, y = mean_TPR)) +
````

  geom_ribbon(aes(ymin = lower_TPR, ymax = upper_TPR),
              alpha = 0.2, fill = "blue", color = NA) +
  geom_line(color = "blue", linewidth = 1.5) +
  geom_abline(slope = 1, intercept = 0, linetype = "dashed", color = "red", linewidth = 1) +
  labs(title = paste("Threshold-Averaged Cross-Validated ROC Curve\n(AUC =", round(cv_auc, 3), ")"),
       x = "False Positive Rate (1 - Specificity)",
       y = "True Positive Rate (Sensitivity)") +
  theme_minimal() +
  theme(plot.title = element_text(hjust = 0.5, size = 14),
        axis.title = element_text(size = 12),
        axis.text = element_text(size = 10)) +
  coord_equal(ratio = 1) +
  scale_x_continuous(limits = c(0, 1), expand = c(0, 0)) +
  scale_y_continuous(limits = c(0, 1), expand = c(0, 0))

print(threshold_roc_plot)

#storing the current plot
ROCplots[[modelcounter]] = threshold_roc_plot
```

#### Importance of variables for the estimated model

The best model was achieved with alpha = `r cv_alpha`, and lambda = `r cv_lambda`.

Below is a plot of the relative importance of the `r ifelse(used_coef <=20, used_coef,20)` most relevant variables.

```{r, echo=FALSE, warning=FALSE, message=FALSE, fig.align='center'}
temp1=varImp(model_reduced)
temp2=sort(as.matrix(temp1$importance),decreasing = TRUE,index.return = TRUE)
if (used_coef<=20){
  plot(temp1,top = used_coef)
} else {
  plot(temp1,top = 20)
}

```

Table of the most important variables for the estimated model

```{r, echo=FALSE, warning=FALSE, message=FALSE, fig.align='center'}
if (used_coef<=20){
  topvarnames=formula(paste("~ ", paste(row.names(temp1$importance)[temp2$ix[1:used_coef]], collapse= "+")," | Group"))
} else {
  topvarnames=formula(paste("~ ", paste(row.names(temp1$importance)[temp2$ix[1:20]], collapse= "+")," | Group"))
}
table1(topvarnames,dataset_filtered_Mean_SD,overall=FALSE)
```

```{r, echo=FALSE, warning=FALSE, message=FALSE}
#Optional to save the results (all R space) of each model

````
save.image(file = paste("Model_",modelcounter,".RData",sep = ""))
modelcounter = modelcounter + 1 #even if the above line is commented, keep the counter update active for the ROCplots
```

Condition Find during the dual task experiment

```{r, echo=FALSE, warning=FALSE, message=FALSE}
# random number seed
set.seed(1245)

# Fit model to training data
model_reduced = train(Group ~ .,
            data = Aset_Mean_SD,
            tuneGrid = myTuning,
            trControl = myControl,
            preProcess = myPreproc,
            method = "glmnet",
            family = "binomial")

results_reduced=get_best_result(model_reduced)
cv_acc = results_reduced$Accuracy.Accuracy
cv_acc_sd = results_reduced$Accuracy.AccuracySD
cv_alpha = results_reduced$alpha
cv_lambda = results_reduced$lambda
if (sum(model_reduced$finalModel$lambda < results[[2]])==0) {
  used_coef=model_reduced$finalModel$df[length(model_reduced$finalModel$df)]
}else{
  used_coef=model_reduced$finalModel$df[which(model_reduced$finalModel$lambda < results_reduced[[2]])[[1]]]
}
cv_auc <- results_reduced$ROC  # If you have twoClassSummary enabled
cv_auc_sd <- results_reduced$ROCSD
```

Using data from Find condition of the dual task experiment, the performance of the model was: Accuracy = `r round(cv_acc, 2)` $\pm$ `r round(cv_acc_sd,2)`.

Cross-validated AUC = `r round(cv_auc,2)` $\pm$ `r round(cv_auc_sd,2)`.

The ROC curve is presented below.

```{r, echo=FALSE, warning=FALSE, message=FALSE, fig.align='center'}
### Generating Threshold-Averaged ROC curve
# Get predictions for best hyperparameters
best_preds <- model_reduced$pred %>%
  filter(alpha == cv_alpha & lambda == cv_lambda)

# Create common FPR grid for threshold averaging
fpr_grid <- seq(0, 1, length.out = 100)

# Calculate TPR at each FPR point for each fold
tpr_matrix <- matrix(NA, nrow = length(fpr_grid), ncol = length(unique(best_preds$Resample)))
folds <- unique(best_preds$Resample)
````

```
for(i in seq_along(folds)) {
  fold_data <- best_preds %>% filter(Resample == folds[i])
  roc_fold <- roc(fold_data$obs, fold_data$EM)

  # Get TPR at each FPR point using interpolation
  tpr_matrix[, i] <- approx(x = 1 - roc_fold$specificities,
                            y = roc_fold$sensitivities,
                            xout = fpr_grid,
                            method = "linear",
                            rule = 2)$y
}

# Calculate mean and SD of TPR across folds at each FPR point
threshold_avg_roc <- data.frame(
  FPR = fpr_grid,
  mean_TPR = rowMeans(tpr_matrix, na.rm = TRUE),
  sd_TPR = apply(tpr_matrix, 1, sd, na.rm = TRUE)
) %>%
  mutate(
    upper_TPR = pmin(mean_TPR + sd_TPR, 1),
    lower_TPR = pmax(mean_TPR - sd_TPR, 0)
  )

# Plot threshold-averaged ROC curve
threshold_roc_plot <- ggplot(threshold_avg_roc, aes(x = FPR, y = mean_TPR)) +
  geom_ribbon(aes(ymin = lower_TPR, ymax = upper_TPR),
              alpha = 0.2, fill = "blue", color = NA) +
  geom_line(color = "blue", linewidth = 1.5) +
  geom_abline(slope = 1, intercept = 0, linetype = "dashed", color = "red", linewidth = 1) +
  labs(title = paste("Threshold-Averaged Cross-Validated ROC Curve\n(AUC =", round(cv_a
uc, 3), ")"),
       x = "False Positive Rate (1 - Specificity)",
       y = "True Positive Rate (Sensitivity)") +
  theme_minimal() +
  theme(plot.title = element_text(hjust = 0.5, size = 14),
        axis.title = element_text(size = 12),
        axis.text = element_text(size = 10)) +
  coord_equal(ratio = 1) +
  scale_x_continuous(limits = c(0, 1), expand = c(0, 0)) +
  scale_y_continuous(limits = c(0, 1), expand = c(0, 0))

print(threshold_roc_plot)

#storing the current plot
ROCplots[[modelcounter]] = threshold_roc_plot
```

Importance of variables for the estimated model

The best model was achieved with alpha = `r cv_alpha`, and lambda = `r cv_lambda`.

Below is a plot of the relative importance of the `r ifelse(used_coef <=20, used_coef,20)` mo
st relevant variables.

````
```{r, echo=FALSE, warning=FALSE, message=FALSE, fig.align='center'}
temp1=varImp(model_reduced)
temp2=sort(as.matrix(temp1$importance),decreasing = TRUE,index.return = TRUE)
if (used_coef<=20){
  plot(temp1,top = used_coef)
} else {
  plot(temp1,top = 20)
}

```

#### Table of the most important variables for the estimated model

```{r, echo=FALSE, warning=FALSE, message=FALSE, fig.align='center'}
if (used_coef<=20){
  topvarnames=formula(paste("~ ", paste(row.names(temp1$importance)[temp2$ix[1:used_c
oef]], collapse= "+")," | Group"))
} else {
  topvarnames=formula(paste("~ ", paste(row.names(temp1$importance)[temp2$ix[1:20]], co
llapse= "+")," | Group"))
}
table1(topvarnames,dataset_filtered_Mean_SD,overall=FALSE)
```

```{r, echo=FALSE, warning=FALSE, message=FALSE}
#Optional to save the results (all R space) of each model
save.image(file = paste("Model_",modelcounter,".RData",sep = ""))
modelcounter = modelcounter + 1 #even if the above line is commented, keep the counter u
pdate active for the ROCplots
```

### Condition Remember during the dual-task experiment

```{r, echo=FALSE, warning=FALSE, message=FALSE}
# random number seed
set.seed(1245)

# Fit model to training data
model_reduced = train(Group ~ .,
            data = Lset_Mean_SD,
            tuneGrid = myTuning,
            trControl = myControl,
            preProcess = myPreproc,
            method = "glmnet",
            family = "binomial")

results_reduced=get_best_result(model_reduced)
cv_acc = results_reduced$Accuracy.Accuracy
cv_acc_sd = results_reduced$Accuracy.AccuracySD
cv_alpha = results_reduced$alpha
cv_lambda = results_reduced$lambda
if (sum(model_reduced$finalModel$lambda < results[[2]])==0) {
  used_coef=model_reduced$finalModel$df[length(model_reduced$finalModel$df)]
}else{
````
````

````
  used_coef=model_reduced$finalModel$df[which(model_reduced$finalModel$lambda < res
ults_reduced[[2]])[[1]]]
}
cv_auc <- results_reduced$ROC  # If you have twoClassSummary enabled
cv_auc_sd <- results_reduced$ROCSD
```
````

Using data from Remember condition of the dual task experiment, the performance of the m odel improved was: Accuracy = `r round(cv_acc, 2)` $\pm$ `r round(cv_acc_sd,2)`.

Cross-validated AUC = `r round(cv_auc,2)` $\pm$ `r round(cv_auc_sd,2)`.

The ROC curve is presented below.

````
```{r, echo=FALSE, warning=FALSE, message=FALSE, fig.align='center'}
### Generating Threshold-Averaged ROC curve
# Get predictions for best hyperparameters
best_preds <- model_reduced$pred %>%
  filter(alpha == cv_alpha & lambda == cv_lambda)

# Create common FPR grid for threshold averaging
fpr_grid <- seq(0, 1, length.out = 100)

# Calculate TPR at each FPR point for each fold
tpr_matrix <- matrix(NA, nrow = length(fpr_grid), ncol = length(unique(best_preds$Resample
)))
folds <- unique(best_preds$Resample)

for(i in seq_along(folds)) {
  fold_data <- best_preds %>% filter(Resample == folds[i])
  roc_fold <- roc(fold_data$obs, fold_data$EM)

  # Get TPR at each FPR point using interpolation
  tpr_matrix[, i] <- approx(x = 1 - roc_fold$specificities,
                         y = roc_fold$sensitivities,
                         xout = fpr_grid,
                         method = "linear",
                         rule = 2)$y
}

# Calculate mean and SD of TPR across folds at each FPR point
threshold_avg_roc <- data.frame(
  FPR = fpr_grid,
  mean_TPR = rowMeans(tpr_matrix, na.rm = TRUE),
  sd_TPR = apply(tpr_matrix, 1, sd, na.rm = TRUE)
) %>%
  mutate(
    upper_TPR = pmin(mean_TPR + sd_TPR, 1),
    lower_TPR = pmax(mean_TPR - sd_TPR, 0)
  )

# Plot threshold-averaged ROC curve
threshold_roc_plot <- ggplot(threshold_avg_roc, aes(x = FPR, y = mean_TPR)) +
  geom_ribbon(aes(ymin = lower_TPR, ymax = upper_TPR),
            alpha = 0.2, fill = "blue", color = NA) +
````

  geom_line(color = "blue", linewidth = 1.5) +
  geom_abline(slope = 1, intercept = 0, linetype = "dashed", color = "red", linewidth = 1) +
  labs(title = paste("Threshold-Averaged Cross-Validated ROC Curve\n(AUC =", round(cv_auc, 3), ")"),
       x = "False Positive Rate (1 - Specificity)",
       y = "True Positive Rate (Sensitivity)") +
  theme_minimal() +
  theme(plot.title = element_text(hjust = 0.5, size = 14),
        axis.title = element_text(size = 12),
        axis.text = element_text(size = 10)) +
  coord_equal(ratio = 1) +
  scale_x_continuous(limits = c(0, 1), expand = c(0, 0)) +
  scale_y_continuous(limits = c(0, 1), expand = c(0, 0))

print(threshold_roc_plot)

#storing the current plot
ROCplots[[modelcounter]] = threshold_roc_plot
```

#### Importance of variables for the estimated model

The best model was achieved with alpha = `r cv_alpha`, and lambda = `r cv_lambda`.

Below is a plot of the relative importance of the `r ifelse(used_coef <=20, used_coef,20)` most relevant variables.

```{r, echo=FALSE, warning=FALSE, message=FALSE, fig.align='center'}
temp1=varImp(model_reduced)
temp2=sort(as.matrix(temp1$importance),decreasing = TRUE,index.return = TRUE)
if (used_coef<=20){
  plot(temp1,top = used_coef)
} else {
  plot(temp1,top = 20)
}

```

Table of the most important variables for the estimated model

```{r, echo=FALSE, warning=FALSE, message=FALSE, fig.align='center'}
if (used_coef<=20){
  topvarnames=formula(paste("~ ", paste(row.names(temp1$importance)[temp2$ix[1:used_coef]], collapse= "+")," | Group"))
} else {
  topvarnames=formula(paste("~ ", paste(row.names(temp1$importance)[temp2$ix[1:20]], collapse= "+")," | Group"))
}
table1(topvarnames,dataset_filtered_Mean_SD,overall=FALSE)
```

```{r, echo=FALSE, warning=FALSE, message=FALSE}
#Optional to save the results (all R space) of each model
save.image(file = paste("Model_",modelcounter,".RData",sep = ""))

```
modelcounter = modelcounter + 1 #even if the above line is commented, keep the counter update active for the ROCplots
```

Classification analysis using SD variables

Further in the post hoc analyses, we decided to check the model performance only using SD variables.

Classification of patients and controls with all conditions together.

We trained the elastic net model to classify participants as being from the Multiple sclerosis or control group using the means of several gait kinematic variables.

The model was trained using a 5-fold cross validation with 5 repetitions and parameter tuning was performed with alpha ranging from 0 to 1 and lambda ranging from 0.05 to 50. The performance metric is classification average accuracy across cross-validation folds. Because the sample size is small, we did not create a separate test set, only validation sets for the cross-validation procedure.

```{r, echo=FALSE, warning=FALSE, message=FALSE}
# random number seed
set.seed(1245)

# Fit model to training data
model = train(Group ~ .,
            data = dataset_filtered_SD,
            tuneGrid = myTuning,
            trControl = myControl,
            preProcess = myPreproc,
            method = "glmnet",
            family = "binomial")

results=get_best_result(model)
cv_acc = results$Accuracy.Accuracy
cv_acc_sd = results$Accuracy.AccuracySD
cv_alpha = results$alpha
cv_lambda = results$lambda
if (sum(model$finalModel$lambda < results[[2]])==0) {
  used_coef=model$finalModel$df[length(model$finalModel$df)]
}else{
  used_coef=model$finalModel$df[which(model$finalModel$lambda < results[[2]])[[1]]]
}
cv_auc <- results$ROC  # If you have twoClassSummary enabled
cv_auc_sd <- results$ROCSD
```

Cross-validation accuracy = `r round(cv_acc,2)` $\pm$ `r round(cv_acc_sd,2)`(mean $\pm$ SD). This means that using `r dim(dataset_filtered_SD)[2]-1` gait kinematic variables (`r (dim(dataset_filtered_SD)[2]-1)/4` in each of the 4 conditions) we are able to classify above chance who is a patient with Multiple Sclerosis and who is a control participant.

Cross-validated AUC = `r round(cv_auc,2)` $\pm$ `r round(cv_auc_sd,2)`.

The ROC curve is presented below.

```{r, echo=FALSE, warning=FALSE, message=FALSE, fig.align='center'}
### Generating Threshold-Averaged ROC curve
# Get predictions for best hyperparameters
best_preds <- model$pred %>%
  filter(alpha == cv_alpha & lambda == cv_lambda)

# Create common FPR grid for threshold averaging
fpr_grid <- seq(0, 1, length.out = 100)

# Calculate TPR at each FPR point for each fold
tpr_matrix <- matrix(NA, nrow = length(fpr_grid), ncol = length(unique(best_preds$Resample
)))
folds <- unique(best_preds$Resample)

for(i in seq_along(folds)) {
  fold_data <- best_preds %>% filter(Resample == folds[i])
  roc_fold <- roc(fold_data$obs, fold_data$EM)

  # Get TPR at each FPR point using interpolation
  tpr_matrix[, i] <- approx(x = 1 - roc_fold$specificities,
                           y = roc_fold$sensitivities,
                           xout = fpr_grid,
                           method = "linear",
                           rule = 2)$y
}

# Calculate mean and SD of TPR across folds at each FPR point
threshold_avg_roc <- data.frame(
  FPR = fpr_grid,
  mean_TPR = rowMeans(tpr_matrix, na.rm = TRUE),
  sd_TPR = apply(tpr_matrix, 1, sd, na.rm = TRUE)
) %>%
  mutate(
    upper_TPR = pmin(mean_TPR + sd_TPR, 1),
    lower_TPR = pmax(mean_TPR - sd_TPR, 0)
  )

# Plot threshold-averaged ROC curve
threshold_roc_plot <- ggplot(threshold_avg_roc, aes(x = FPR, y = mean_TPR)) +
  geom_ribbon(aes(ymin = lower_TPR, ymax = upper_TPR),
              alpha = 0.2, fill = "blue", color = NA) +
  geom_line(color = "blue", linewidth = 1.5) +
  geom_abline(slope = 1, intercept = 0, linetype = "dashed", color = "red", linewidth = 1) +
  labs(title = paste("Threshold-Averaged Cross-Validated ROC Curve\n(AUC =", round(cv_a
uc, 3), ")"),
      x = "False Positive Rate (1 - Specificity)",
      y = "True Positive Rate (Sensitivity)") +
  theme_minimal() +
  theme(plot.title = element_text(hjust = 0.5, size = 14),
        axis.title = element_text(size = 12),
        axis.text = element_text(size = 10)) +
  coord_equal(ratio = 1) +
  scale_x_continuous(limits = c(0, 1), expand = c(0, 0)) +
  scale_y_continuous(limits = c(0, 1), expand = c(0, 0))
```

print(threshold_roc_plot)

#storing the current plot
ROCplots[[modelcounter]] = threshold_roc_plot
```

### Importance of variables for the estimated model

The best model was achieved with alpha = `r cv_alpha`, and lambda = `r cv_lambda`

Below is a plot of the relative importance of the `r ifelse(used_coef <=20, used_coef,20)` most relevant variables.

```{r, echo=FALSE, warning=FALSE, message=FALSE, fig.align='center'}
temp1=varImp(model)
temp2=sort(as.matrix(temp1$importance),decreasing = TRUE,index.return = TRUE)
if (used_coef<=20){
  plot(temp1,top = used_coef)
} else {
  plot(temp1,top = 20)
}

```

Table of the most important variables for the estimated model

```{r, echo=FALSE, warning=FALSE, message=FALSE, fig.align='center'}
if (used_coef<=20){
  topvarnames=formula(paste("~ ", paste(row.names(temp1$importance)[temp2$ix[1:used_coef]], collapse= "+")," | Group"))
} else {
  topvarnames=formula(paste("~ ", paste(row.names(temp1$importance)[temp2$ix[1:20]], collapse= "+")," | Group"))
}
table1(topvarnames,dataset_filtered_SD,overall=FALSE)
```

```{r, echo=FALSE, warning=FALSE, message=FALSE}
#Optional to save the results (all R space) of each model
save.image(file = paste("Model_",modelcounter,".RData",sep = ""))
modelcounter = modelcounter + 1 #even if the above line is commented, keep the counter update active for the ROCplots
```

Classification in each condition separately

After the analysis with all conditions, we decided to explore each experimental condition separately. Legend of the experimental conditions: F = Free Walk (single task experiment);C = Click-Walk (Walk in the dual task n-back experiment) A = 0-back (during dual task n-back experiment); and L = 2-back (during the dual-task n-back experiment).

Condition Free Walk during single task experiment
```

````
```{r, echo=FALSE, warning=FALSE, message=FALSE}
# random number seed
set.seed(1245)

# Fit model to training data
model_reduced = train(Group ~ .,
            data = Fset_SD,
            tuneGrid = myTuning,
            trControl = myControl,
            preProcess = myPreproc,
            method = "glmnet",
            family = "binomial")

results_reduced=get_best_result(model_reduced)
cv_acc = results_reduced$Accuracy.Accuracy
cv_acc_sd = results_reduced$Accuracy.AccuracySD
cv_alpha = results_reduced$alpha
cv_lambda = results_reduced$lambda
if (sum(model_reduced$finalModel$lambda < results[[2]])==0) {
  used_coef=model_reduced$finalModel$df[length(model_reduced$finalModel$df)]
}else{
  used_coef=model_reduced$finalModel$df[which(model_reduced$finalModel$lambda < results_reduced[[2]])[[1]]]
}
cv_auc <- results_reduced$ROC  # If you have twoClassSummary enabled
cv_auc_sd <- results_reduced$ROCSD
```
````

Using data from free walk condition only, the performance of the model was still very good: Accuracy = `r round(cv_acc, 2)` $\pm$ `r round(cv_acc_sd,2)`.

Cross-validated AUC = `r round(cv_auc,2)` $\pm$ `r round(cv_auc_sd,2)`.

The ROC curve is presented below.

````
```{r, echo=FALSE, warning=FALSE, message=FALSE, fig.align='center'}
### Generating Threshold-Averaged ROC curve
# Get predictions for best hyperparameters
best_preds <- model_reduced$pred %>%
  filter(alpha == cv_alpha & lambda == cv_lambda)

# Create common FPR grid for threshold averaging
fpr_grid <- seq(0, 1, length.out = 100)

# Calculate TPR at each FPR point for each fold
tpr_matrix <- matrix(NA, nrow = length(fpr_grid), ncol = length(unique(best_preds$Resample)))
folds <- unique(best_preds$Resample)

for(i in seq_along(folds)) {
  fold_data <- best_preds %>% filter(Resample == folds[i])
  roc_fold <- roc(fold_data$obs, fold_data$EM)

  # Get TPR at each FPR point using interpolation
  tpr_matrix[, i] <- approx(x = 1 - roc_fold$specificities,
````
````

```
                        y = roc_fold$sensitivities,
                        xout = fpr_grid,
                        method = "linear",
                        rule = 2)$y
}

# Calculate mean and SD of TPR across folds at each FPR point
threshold_avg_roc <- data.frame(
  FPR = fpr_grid,
  mean_TPR = rowMeans(tpr_matrix, na.rm = TRUE),
  sd_TPR = apply(tpr_matrix, 1, sd, na.rm = TRUE)
) %>%
  mutate(
    upper_TPR = pmin(mean_TPR + sd_TPR, 1),
    lower_TPR = pmax(mean_TPR - sd_TPR, 0)
  )

# Plot threshold-averaged ROC curve
threshold_roc_plot <- ggplot(threshold_avg_roc, aes(x = FPR, y = mean_TPR)) +
  geom_ribbon(aes(ymin = lower_TPR, ymax = upper_TPR),
              alpha = 0.2, fill = "blue", color = NA) +
  geom_line(color = "blue", linewidth = 1.5) +
  geom_abline(slope = 1, intercept = 0, linetype = "dashed", color = "red", linewidth = 1) +
  labs(title = paste("Threshold-Averaged Cross-Validated ROC Curve\n(AUC =", round(cv_auc, 3), ")"),
       x = "False Positive Rate (1 - Specificity)",
       y = "True Positive Rate (Sensitivity)") +
  theme_minimal() +
  theme(plot.title = element_text(hjust = 0.5, size = 14),
        axis.title = element_text(size = 12),
        axis.text = element_text(size = 10)) +
  coord_equal(ratio = 1) +
  scale_x_continuous(limits = c(0, 1), expand = c(0, 0)) +
  scale_y_continuous(limits = c(0, 1), expand = c(0, 0))

print(threshold_roc_plot)

#storing the current plot
ROCplots[[modelcounter]] = threshold_roc_plot
```

Importance of variables for the estimated model

The best model was achieved with alpha = `r cv_alpha`, and lambda = `r cv_lambda`.

Below is a plot of the relative importance of the `r ifelse(used_coef <=20, used_coef,20)` most relevant variables.

```{r, echo=FALSE, warning=FALSE, message=FALSE, fig.align='center'}
temp1=varImp(model_reduced)
temp2=sort(as.matrix(temp1$importance),decreasing = TRUE,index.return = TRUE)
if (used_coef<=20){
  plot(temp1,top = used_coef)
} else {
  plot(temp1,top = 20)
```

}

```

#### Table of the most important variables for the estimated model

```{r, echo=FALSE, warning=FALSE, message=FALSE, fig.align='center'}
if (used_coef<=20){
  topvarnames=formula(paste("~ ", paste(row.names(temp1$importance)[temp2$ix[1:used_c
oef]], collapse= "+")," | Group"))
} else {
  topvarnames=formula(paste("~ ", paste(row.names(temp1$importance)[temp2$ix[1:20]], co
llapse= "+")," | Group"))
}
table1(topvarnames,dataset_filtered_SD,overall=FALSE)
```

```{r, echo=FALSE, warning=FALSE, message=FALSE}
#Optional to save the results (all R space) of each model
save.image(file = paste("Model_",modelcounter,".RData",sep = ""))
modelcounter = modelcounter + 1 #even if the above line is commented, keep the counter u
pdate active for the ROCplots
```

Condition Walk during the dual task experiment

```{r, echo=FALSE, warning=FALSE, message=FALSE}
# random number seed
set.seed(1245)

# Fit model to training data
model_reduced = train(Group ~ .,
            data = Cset_SD,
            tuneGrid = myTuning,
            trControl = myControl,
            preProcess = myPreproc,
            method = "glmnet",
            family = "binomial")

results_reduced=get_best_result(model_reduced)
cv_acc = results_reduced$Accuracy.Accuracy
cv_acc_sd = results_reduced$Accuracy.AccuracySD
cv_alpha = results_reduced$alpha
cv_lambda = results_reduced$lambda
if (sum(model_reduced$finalModel$lambda < results[[2]])==0) {
  used_coef=model_reduced$finalModel$df[length(model_reduced$finalModel$df)]
}else{
  used_coef=model_reduced$finalModel$df[which(model_reduced$finalModel$lambda < res
ults_reduced[[2]])[[1]]]
}
cv_auc <- results_reduced$ROC  # If you have twoClassSummary enabled
cv_auc_sd <- results_reduced$ROCSD
```
```

Using data from walk condition of the dual task experiment, the performance of the model w as: Accuracy = `r round(cv_acc, 2)` $\pm$ `r round(cv_acc_sd,2)`.

Cross-validated AUC = `r round(cv_auc,2)` $\pm$ `r round(cv_auc_sd,2)`.

The ROC curve is presented below.

```
```{r, echo=FALSE, warning=FALSE, message=FALSE, fig.align='center'}
### Generating Threshold-Averaged ROC curve
# Get predictions for best hyperparameters
best_preds <- model_reduced$pred %>%
  filter(alpha == cv_alpha & lambda == cv_lambda)

# Create common FPR grid for threshold averaging
fpr_grid <- seq(0, 1, length.out = 100)

# Calculate TPR at each FPR point for each fold
tpr_matrix <- matrix(NA, nrow = length(fpr_grid), ncol = length(unique(best_preds$Resample
)))
folds <- unique(best_preds$Resample)

for(i in seq_along(folds)) {
  fold_data <- best_preds %>% filter(Resample == folds[i])
  roc_fold <- roc(fold_data$obs, fold_data$EM)

  # Get TPR at each FPR point using interpolation
  tpr_matrix[, i] <- approx(x = 1 - roc_fold$specificities,
                           y = roc_fold$sensitivities,
                           xout = fpr_grid,
                           method = "linear",
                           rule = 2)$y
}

# Calculate mean and SD of TPR across folds at each FPR point
threshold_avg_roc <- data.frame(
  FPR = fpr_grid,
  mean_TPR = rowMeans(tpr_matrix, na.rm = TRUE),
  sd_TPR = apply(tpr_matrix, 1, sd, na.rm = TRUE)
) %>%
  mutate(
    upper_TPR = pmin(mean_TPR + sd_TPR, 1),
    lower_TPR = pmax(mean_TPR - sd_TPR, 0)
  )

# Plot threshold-averaged ROC curve
threshold_roc_plot <- ggplot(threshold_avg_roc, aes(x = FPR, y = mean_TPR)) +
  geom_ribbon(aes(ymin = lower_TPR, ymax = upper_TPR),
            alpha = 0.2, fill = "blue", color = NA) +
  geom_line(color = "blue", linewidth = 1.5) +
  geom_abline(slope = 1, intercept = 0, linetype = "dashed", color = "red", linewidth = 1) +
  labs(title = paste("Threshold-Averaged Cross-Validated ROC Curve\n(AUC =", round(cv_a
uc, 3), ")"),
      x = "False Positive Rate (1 - Specificity)",
      y = "True Positive Rate (Sensitivity)") +
  theme_minimal() +
```
```

  theme(plot.title = element_text(hjust = 0.5, size = 14),
        axis.title = element_text(size = 12),
        axis.text = element_text(size = 10)) +
  coord_equal(ratio = 1) +
  scale_x_continuous(limits = c(0, 1), expand = c(0, 0)) +
  scale_y_continuous(limits = c(0, 1), expand = c(0, 0))

print(threshold_roc_plot)

#storing the current plot
ROCplots[[modelcounter]] = threshold_roc_plot
```

#### Importance of variables for the estimated model

The best model was achieved with alpha = `r cv_alpha`, and lambda = `r cv_lambda`, which
corresponds to `r round(100*model_reduced$finalModel$dev.ratio[which(model_reduced$fin
alModel$lambda < results_reduced[[2]])[[1]]],2)`% of explained deviance, using `r used_coef`
variables

Below is a plot of the relative importance of the `r ifelse(used_coef <=20, used_coef,20)` mo
st relevant variables.

```{r, echo=FALSE, warning=FALSE, message=FALSE, fig.align='center'}
temp1=varImp(model_reduced)
temp2=sort(as.matrix(temp1$importance),decreasing = TRUE,index.return = TRUE)
if (used_coef<=20){
  plot(temp1,top = used_coef)
} else {
  plot(temp1,top = 20)
}

```

Table of the most important variables for the estimated model

```{r, echo=FALSE, warning=FALSE, message=FALSE, fig.align='center'}
if (used_coef<=20){
  topvarnames=formula(paste("~ ", paste(row.names(temp1$importance)[temp2$ix[1:used_c
oef]], collapse= "+")," | Group"))
} else {
  topvarnames=formula(paste("~ ", paste(row.names(temp1$importance)[temp2$ix[1:20]], co
llapse= "+")," | Group"))
}
table1(topvarnames,dataset_filtered_SD,overall=FALSE)
```

```{r, echo=FALSE, warning=FALSE, message=FALSE}
#Optional to save the results (all R space) of each model
save.image(file = paste("Model_",modelcounter,".RData",sep = ""))
modelcounter = modelcounter + 1 #even if the above line is commented, keep the counter u
pdate active for the ROCplots
```

Condition Find during the dual task experiment
```

````
```{r, echo=FALSE, warning=FALSE, message=FALSE}
# random number seed
set.seed(1245)

# Fit model to training data
model_reduced = train(Group ~ .,
             data = Aset_SD,
             tuneGrid = myTuning,
             trControl = myControl,
             preProcess = myPreproc,
             method = "glmnet",
             family = "binomial")

results_reduced=get_best_result(model_reduced)
cv_acc = results_reduced$Accuracy.Accuracy
cv_acc_sd = results_reduced$Accuracy.AccuracySD
cv_alpha = results_reduced$alpha
cv_lambda = results_reduced$lambda
if (sum(model_reduced$finalModel$lambda < results[[2]])==0) {
  used_coef=model_reduced$finalModel$df[length(model_reduced$finalModel$df)]
}else{
  used_coef=model_reduced$finalModel$df[which(model_reduced$finalModel$lambda < res
ults_reduced[[2]])[[1]]]
}
cv_auc <- results_reduced$ROC  # If you have twoClassSummary enabled
cv_auc_sd <- results_reduced$ROCSD
```
````

Using data from Find condition of the dual task experiment, the performance of the model wa
s: Accuracy = `r round(cv_acc, 2)` $\pm$ `r round(cv_acc_sd,2)`.

Cross-validated AUC = `r round(cv_auc,2)` $\pm$ `r round(cv_auc_sd,2)`.

The ROC curve is presented below.

````
```{r, echo=FALSE, warning=FALSE, message=FALSE, fig.align='center'}
### Generating Threshold-Averaged ROC curve
# Get predictions for best hyperparameters
best_preds <- model_reduced$pred %>%
  filter(alpha == cv_alpha & lambda == cv_lambda)

# Create common FPR grid for threshold averaging
fpr_grid <- seq(0, 1, length.out = 100)

# Calculate TPR at each FPR point for each fold
tpr_matrix <- matrix(NA, nrow = length(fpr_grid), ncol = length(unique(best_preds$Resample
)))
folds <- unique(best_preds$Resample)

for(i in seq_along(folds)) {
  fold_data <- best_preds %>% filter(Resample == folds[i])
  roc_fold <- roc(fold_data$obs, fold_data$EM)

  # Get TPR at each FPR point using interpolation
````
````

````
  tpr_matrix[, i] <- approx(x = 1 - roc_fold$specificities,
                         y = roc_fold$sensitivities,
                         xout = fpr_grid,
                         method = "linear",
                         rule = 2)$y
}

# Calculate mean and SD of TPR across folds at each FPR point
threshold_avg_roc <- data.frame(
  FPR = fpr_grid,
  mean_TPR = rowMeans(tpr_matrix, na.rm = TRUE),
  sd_TPR = apply(tpr_matrix, 1, sd, na.rm = TRUE)
) %>%
  mutate(
    upper_TPR = pmin(mean_TPR + sd_TPR, 1),
    lower_TPR = pmax(mean_TPR - sd_TPR, 0)
  )

# Plot threshold-averaged ROC curve
threshold_roc_plot <- ggplot(threshold_avg_roc, aes(x = FPR, y = mean_TPR)) +
  geom_ribbon(aes(ymin = lower_TPR, ymax = upper_TPR),
             alpha = 0.2, fill = "blue", color = NA) +
  geom_line(color = "blue", linewidth = 1.5) +
  geom_abline(slope = 1, intercept = 0, linetype = "dashed", color = "red", linewidth = 1) +
  labs(title = paste("Threshold-Averaged Cross-Validated ROC Curve\n(AUC =", round(cv_a
uc, 3), ")"),
       x = "False Positive Rate (1 - Specificity)",
       y = "True Positive Rate (Sensitivity)") +
  theme_minimal() +
  theme(plot.title = element_text(hjust = 0.5, size = 14),
        axis.title = element_text(size = 12),
        axis.text = element_text(size = 10)) +
  coord_equal(ratio = 1) +
  scale_x_continuous(limits = c(0, 1), expand = c(0, 0)) +
  scale_y_continuous(limits = c(0, 1), expand = c(0, 0))

print(threshold_roc_plot)

#storing the current plot
ROCplots[[modelcounter]] = threshold_roc_plot
```

#### Importance of variables for the estimated model

The best model was achieved with alpha = `r cv_alpha`, and lambda = `r cv_lambda`.

Below is a plot of the relative importance of the `r ifelse(used_coef <=20, used_coef,20)` mo
st relevant variables.

```{r, echo=FALSE, warning=FALSE, message=FALSE, fig.align='center'}
temp1=varImp(model_reduced)
temp2=sort(as.matrix(temp1$importance),decreasing = TRUE,index.return = TRUE)
if (used_coef<=20){
  plot(temp1,top = used_coef)
} else {
````

  plot(temp1,top = 20)
}

```

#### Table of the most important variables for the estimated model

```{r, echo=FALSE, warning=FALSE, message=FALSE, fig.align='center'}
if (used_coef<=20){
  topvarnames=formula(paste("~ ", paste(row.names(temp1$importance)[temp2$ix[1:used_coef]], collapse= "+")," | Group"))
} else {
  topvarnames=formula(paste("~ ", paste(row.names(temp1$importance)[temp2$ix[1:20]], collapse= "+")," | Group"))
}
table1(topvarnames,dataset_filtered_SD,overall=FALSE)
```

```{r, echo=FALSE, warning=FALSE, message=FALSE}
#Optional to save the results (all R space) of each model
save.image(file = paste("Model_",modelcounter,".RData",sep = ""))
modelcounter = modelcounter + 1 #even if the above line is commented, keep the counter update active for the ROCplots
```

Condition Remember during the dual-task experiment

```{r, echo=FALSE, warning=FALSE, message=FALSE}
# random number seed
set.seed(1245)

# Fit model to training data
model_reduced = train(Group ~ .,
             data = Lset_SD,
             tuneGrid = myTuning,
             trControl = myControl,
             preProcess = myPreproc,
             method = "glmnet",
             family = "binomial")

results_reduced=get_best_result(model_reduced)
cv_acc = results_reduced$Accuracy.Accuracy
cv_acc_sd = results_reduced$Accuracy.AccuracySD
cv_alpha = results_reduced$alpha
cv_lambda = results_reduced$lambda
if (sum(model_reduced$finalModel$lambda < results[[2]])==0) {
  used_coef=model_reduced$finalModel$df[length(model_reduced$finalModel$df)]
}else{
  used_coef=model_reduced$finalModel$df[which(model_reduced$finalModel$lambda < results_reduced[[2]])[[1]]]
}
cv_auc <- results_reduced$ROC  # If you have twoClassSummary enabled
cv_auc_sd <- results_reduced$ROCSD
```
```

Using data from Remember condition of the dual task experiment, the performance of the model improved was: Accuracy = `r round(cv_acc, 2)` $\pm$ `r round(cv_acc_sd,2)`.

Cross-validated AUC = `r round(cv_auc,2)` $\pm$ `r round(cv_auc_sd,2)`.

The ROC curve is presented below.

````
```{r, echo=FALSE, warning=FALSE, message=FALSE, fig.align='center'}
### Generating Threshold-Averaged ROC curve
# Get predictions for best hyperparameters
best_preds <- model_reduced$pred %>%
  filter(alpha == cv_alpha & lambda == cv_lambda)

# Create common FPR grid for threshold averaging
fpr_grid <- seq(0, 1, length.out = 100)

# Calculate TPR at each FPR point for each fold
tpr_matrix <- matrix(NA, nrow = length(fpr_grid), ncol = length(unique(best_preds$Resample
)))
folds <- unique(best_preds$Resample)

for(i in seq_along(folds)) {
  fold_data <- best_preds %>% filter(Resample == folds[i])
  roc_fold <- roc(fold_data$obs, fold_data$EM)

  # Get TPR at each FPR point using interpolation
  tpr_matrix[, i] <- approx(x = 1 - roc_fold$specificities,
                            y = roc_fold$sensitivities,
                            xout = fpr_grid,
                            method = "linear",
                            rule = 2)$y
}

# Calculate mean and SD of TPR across folds at each FPR point
threshold_avg_roc <- data.frame(
  FPR = fpr_grid,
  mean_TPR = rowMeans(tpr_matrix, na.rm = TRUE),
  sd_TPR = apply(tpr_matrix, 1, sd, na.rm = TRUE)
) %>%
  mutate(
    upper_TPR = pmin(mean_TPR + sd_TPR, 1),
    lower_TPR = pmax(mean_TPR - sd_TPR, 0)
  )

# Plot threshold-averaged ROC curve
threshold_roc_plot <- ggplot(threshold_avg_roc, aes(x = FPR, y = mean_TPR)) +
  geom_ribbon(aes(ymin = lower_TPR, ymax = upper_TPR),
              alpha = 0.2, fill = "blue", color = NA) +
  geom_line(color = "blue", linewidth = 1.5) +
  geom_abline(slope = 1, intercept = 0, linetype = "dashed", color = "red", linewidth = 1) +
  labs(title = paste("Threshold-Averaged Cross-Validated ROC Curve\n(AUC =", round(cv_a
uc, 3), ")"),
       x = "False Positive Rate (1 - Specificity)",
       y = "True Positive Rate (Sensitivity)") +
  theme_minimal() +
````
````

  theme(plot.title = element_text(hjust = 0.5, size = 14),
        axis.title = element_text(size = 12),
        axis.text = element_text(size = 10)) +
  coord_equal(ratio = 1) +
  scale_x_continuous(limits = c(0, 1), expand = c(0, 0)) +
  scale_y_continuous(limits = c(0, 1), expand = c(0, 0))

print(threshold_roc_plot)

#storing the current plot
ROCplots[[modelcounter]] = threshold_roc_plot
```

#### Importance of variables for the estimated model

The best model was achieved with alpha = `r cv_alpha`, and lambda = `r cv_lambda`.

Below is a plot of the relative importance of the `r ifelse(used_coef <=20, used_coef,20)` most relevant variables.

```{r, echo=FALSE, warning=FALSE, message=FALSE, fig.align='center'}
temp1=varImp(model_reduced)
temp2=sort(as.matrix(temp1$importance),decreasing = TRUE,index.return = TRUE)
if (used_coef<=20){
  plot(temp1,top = used_coef)
} else {
  plot(temp1,top = 20)
}

```

Table of the most important variables for the estimated model

```{r, echo=FALSE, warning=FALSE, message=FALSE, fig.align='center'}
if (used_coef<=20){
  topvarnames=formula(paste("~ ", paste(row.names(temp1$importance)[temp2$ix[1:used_coef]], collapse= "+")," | Group"))
} else {
  topvarnames=formula(paste("~ ", paste(row.names(temp1$importance)[temp2$ix[1:20]], collapse= "+")," | Group"))
}
table1(topvarnames,dataset_filtered_SD,overall=FALSE)
```

```{r, echo=FALSE, warning=FALSE, message=FALSE}
#Optional to save the results (all R space) of each model
save.image(file = paste("Model_",modelcounter,".RData",sep = ""))
modelcounter = modelcounter + 1 #even if the above line is commented, keep the counter update active for the ROCplots
```

```{r, echo=FALSE, warning=FALSE, message=FALSE, fig.width=16, fig.height=19}
# Arrange in grid
orderedROCs=ROCplots
orderedROCs[[1]]=ROCplots[[1]]

```
orderedROCs[[2]]=ROCplots[[6]]
orderedROCs[[3]]=ROCplots[[11]]
orderedROCs[[4]]=ROCplots[[2]]
orderedROCs[[5]]=ROCplots[[7]]
orderedROCs[[6]]=ROCplots[[12]]
orderedROCs[[7]]=ROCplots[[3]]
orderedROCs[[8]]=ROCplots[[8]]
orderedROCs[[9]]=ROCplots[[13]]
orderedROCs[[10]]=ROCplots[[4]]
orderedROCs[[11]]=ROCplots[[9]]
orderedROCs[[12]]=ROCplots[[14]]
orderedROCs[[13]]=ROCplots[[5]]
orderedROCs[[14]]=ROCplots[[10]]
orderedROCs[[15]]=ROCplots[[15]]

row_names <- c("All Conditions", "Free walk", "Click-walk", "0-back", "2-back")
col_names <- c("Means", "Means + SDs", "SDs")

# Create row labels (rotate 90 degrees)
row_labels <- lapply(row_names, function(x) {
  textGrob(x, rot = 90, gp = gpar(fontsize = 14, fontface = "bold"))
})

# Create column labels
col_labels <- lapply(col_names, function(x) {
  textGrob(x, gp = gpar(fontsize = 14, fontface = "bold"))
})

# Create the layout
final_plot <- arrangeGrob(
  # Empty top-left corner + column titles
  arrangeGrob(
    rectGrob(gp = gpar(col = NA)),  # Empty corner
    arrangeGrob(grobs = col_labels, nrow = 1),
    nrow = 1, widths = c(0.08, 0.92)
  ),

  # Row labels + main plots
  arrangeGrob(
    arrangeGrob(grobs = row_labels, ncol = 1),
    arrangeGrob(grobs = orderedROCs, ncol = 3, nrow = 5),
    ncol = 2, widths = c(0.04, 0.96)
  ),

  nrow = 2, heights = c(0.05, 0.95),

  # Global titles
  top = textGrob("ROC curves of elastic net models",
                gp = gpar(fontsize = 16, fontface = "bold")))

grid.draw(final_plot)

# ## Saving the plot
# # Set up high-resolution PNG
# png("roc_curves_combined.png",
```

~~~
#     width = 16, height = 19, units = "in", res = 1200, bg = "white")
#
# # Draw your plot
# grid.draw(final_plot)
#
# # Close the device
# dev.off()
```

```{r, echo=FALSE, warning=FALSE, message=FALSE}
## Stop parallel processing:
stopCluster(cl)
~~~
